# Predictive interception across speed profiles

**DOI:** 10.3389/fncir.2026.1848603

**Published:** 2026-07-13

**Authors:** Inmaculada Márquez, Mario Treviño

**Affiliations:** 1 Laboratorio De Plasticidad Cortical y Aprendizaje Perceptual, Instituto De Neurociencias, Universidad De Guadalajara, Jalisco, Mexico; 2 Departamento De Psicología, Centro Universitario De La Ciénega, Universidad De GuadaLajara, Ocotlán, Mexico; 3 Laboratorio De Neurofisiología, Departamento De Bioingeniería Traslacional, Centro Universitario De Ciencias Exactas e Ingenierías, Jalisco, Mexico

**Keywords:** eye tracking, sensory prediction errors, target acceleration, target speed, visual masking, visuomotor control

## Abstract

Visuomotor interception requires predicting a moving target’s future state to compensate for sensorimotor delays. Predictive control has been studied mainly under constant-speed motion. How observers adapt when the temporal structure of target speed (*v*_*T*_) varies remains unclear. We examined interception across *v*_*T*_ profiles within the same individuals: constant, linear-ramp, and nonlinear (sinusoidal and semicircular). Participants intercepted a moving target while eye and hand movements were recorded; on a subset of trials, visual feedback was removed after an observation period to force reliance on internal estimates. Behavior stayed strongly dependent on *v*_*T*_ even under transient visual loss, but this dependence was constrained by how *v*_*T*_ evolved over time. Removing feedback increased spatial error and gaze–target distance, with modest effects at constant and linear *v*_*T*_ and larger errors at nonlinear *v*_*T*_. Speed matching stayed stable, indicating that local velocity estimates are preserved but insufficient to prevent cumulative spatial error under more complex dynamics. Oculomotor measures were more sensitive than manual control, suggesting partially dissociable effectors. A within-cycle matched-velocity test on the linear-ramp data revealed acceleration–deceleration differences at every velocity bin and metric; the representation is velocity-dominated but not velocity-only, and it exploits higher-order kinematic information at least partially. Exploratory pupillometry showed stable pupil size during full visibility and a reduction late in masked trials, with outcome-dependent modulation independent of gaze position at target disappearance. Predictive interception thus relies on velocity-dominated representations that preserve sensitivity to higher-order kinematic information, enabling robust performance across simple motion profiles but imposing limits when target dynamics become nonlinear.

## Introduction

Visuomotor interactions occur under temporal constraints imposed by neural processing delays. Visual transduction, axonal conduction, synaptic transmission, and central processing introduce latencies between sensory events and motor responses that can reach tens to hundreds of milliseconds. In many instances, these delays make purely reactive control insufficient for tasks that require rapid interaction with moving objects (Márquez and Treviño, 2026). Consequently, many theoretical frameworks propose that the nervous system relies on predictive mechanisms that estimate the future state of external objects and the body, allowing motor commands to be “issued” in advance of delayed sensory feedback (Franklin and Wolpert, 2011; Kawato, 1999; Wolpert et al., 1995). In interception tasks, such predictive control allows actions to be directed toward where a target is expected to be, rather than where it is currently perceived (Márquez and Treviño, 2024b; Treviño et al., 2026; Treviño and Márquez, 2025b).

Predictive motor control is commonly described in terms of internal models that generate expectations about the sensory consequences of actions. When incoming sensory input deviates from these expectations, sensory prediction errors, in principle, act as signals that can update ongoing movements and refine internal representations across repeated interactions (Albert et al., 2023; Izawa and Shadmehr, 2011; Shadmehr et al., 2010; Tseng et al., 2007). These error signals have been shown to support both rapid online corrections and slower adaptive recalibration (Kim et al., 2023; Márquez and Treviño, 2025; Schlerf et al., 2012; Tsay et al., 2022). Notably, despite this continuous interaction between predictive estimation and delayed feedback, the visuomotor system maintains stable performance in dynamic environments (Brooks and Cullen, 2013; Körding and Wolpert, 2006; Seidler et al., 2013).

Interception tasks provide a particularly clear context for studying predictive control. Actions such as catching or striking a flying object require movements directed toward the target’s future position rather than its currently perceived location. Humans nonetheless achieve high levels of spatial and temporal precision in such tasks, suggesting that predictive mechanisms operate efficiently to integrate available motion information and continuously update ongoing movements (Brenner and Smeets, 2018; Regan, 1997; Zhao and Warren, 2013).

Across behavioral studies, target speed (*v*_*T*_) has emerged as a key determinant of interceptive behavior. For successful interception, participants scale their movement speed to match the target, and both manual and oculomotor responses track variations in *v*_*T*_ (Brenner and Smeets, 2018; Márquez and Treviño, 2024b; Regan, 1997; Treviño et al., 2024). However, most of these experiments used targets moving at constant velocity. Constant-speed paradigms offer analytical simplicity but provide a limited test of predictive capabilities, because natural object motion rarely remains constant.

In natural environments, motion trajectories typically arise from interactions among forces such as gravity, aerodynamic drag, and surface contact, producing regular variations in speed. A familiar example is the flight of a frisbee: when released with spin and tilt, aerodynamic lift, drag, and gravity jointly shape its trajectory, causing it to rise, slow, and later descend more steeply as these forces act over time (Baumback, 2010). Despite this non-uniform motion, people routinely position themselves to intercept the disc with high accuracy. Two non-exclusive accounts have been offered. One is that observers exploit empirical priors or heuristics about the underlying dynamics (Delle Monache et al., 2023; McCurdy et al., 2025; de Rugy et al., 2012; Slupinski et al., 2018; Treviño and Márquez, 2025a; Zago et al., 2004). The other is that observers rely primarily on the most recent instantaneous speed estimate, particularly when speed changes occur within short viewing intervals (Brouwer et al., 2002; Tresilian, 1999). Neurophysiological evidence broadly supports this picture: neurons in motion-sensitive cortical areas, such as MT, show robust tuning for direction and speed, whereas explicit representations of higher-order motion changes appear less prominent (Tsutsui et al., 2021). Whether predictive interception relies primarily on instantaneous speed representations or whether it also exploits explicit encoding of changes in speed remains open.

Another approach for probing predictive mechanisms is to manipulate the availability of visual feedback. When a moving target becomes occluded, behavior can no longer rely on continuous sensory updates and must instead employ internally generated estimates of target motion (Adams et al., 2012; Maselli et al., 2022). In laboratory occlusion paradigms, predictive eye movements often continue along the expected trajectory of the target, while manual interception tends to degrade more rapidly (Cámara et al., 2018; Kreyenmeier et al., 2021). Gaze samples target motion directly, whereas the hand produces motor responses driven by that visual input; the two operate on different timescales and for different purposes, so dissociations between them depend on task structure and are a measurement to make, not a result to assume. The magnitude of performance degradation during occlusion also depends on the temporal structure of target motion, with more strongly modulated trajectories posing greater challenges than uniformly moving ones (Kreyenmeier et al., 2021, 2022). These observations, however, come from separate paradigms and separate samples. Comparing several temporal forms of *v*_*T*_ within the same observers holds the interceptor constant while only the speed signal changes, thereby isolating the contribution of motion structure from differences between individuals.

Existing evidence, therefore, leaves three connected open questions. First, how strongly does predictive interception depend on the temporal structure of *v*_*T*_, across different speed regimes compared within the same observers? Second, do oculomotor and manual control degrade in the same way when visual feedback is removed, or do they differ in their sensitivity to motion structure? Third, does the cost of occlusion depend on where the mask is placed in the speed cycle? To our knowledge, no prior study has combined within-subject contrasts of several speed regimes with phase-locked masking in a unified design.

The present study addresses these issues by examining visuomotor interception across several *v*_*T*_ regimes while explicitly manipulating visual feedback. Participants intercepted a moving target whose instantaneous speed varied across trials, and on a subset of trials, the target was visually masked after an initial observation period, forcing reliance on internal motion estimates. Continuous eye-tracking and manual interception measures were used to quantify oculomotor tracking and hand control across these motion regimes. This design isolates how the temporal structure of *v*_*T*_ constrains predictive control.

Within the masked trials, we also used phase-locked masking: brief, phase-aligned occlusions placed at controlled points of an oscillatory speed cycle. Unlike continuous occlusion, phase-locked masking probes whether transient visual loss carries a different cost depending on which segment of the speed signal is removed. Together, these manipulations allow direct within-subject contrasts across speed regimes matched for instantaneous speed range.

The design yields two contrasting predictions. If interception relies primarily on local velocity estimates, behavior should be relatively stable when the local-velocity approximation holds. When that approximation breaks down, errors should accumulate once visual input is removed, and should scale with the rate at which speed varies. Phase-locked masking adds a finer-grained prediction. A representation that encodes the sign of acceleration should produce different costs at different cycle phases and should also distinguish acceleration from deceleration at matched instantaneous velocity within an unmasked cycle. The eye–hand comparison adds a third angle: gaze and manual control draw on motion information differently, so the size of any dissociation between them helps locate where the limit on prediction sits.

## Materials and methods

### Participants

160 right-handed adults (82 women) participated across four independent cohorts (E_1_–E_4_; see Experimental Design and Supplementary Table 1). Participants were 18 to 35 years old (mean = 21.7 ± 0.2 years). All participants reported no history of neurological, psychiatric, or neurodevelopmental disorders and no use of psychoactive medication. Visual acuity was verified as normal or corrected to normal using a standard Snellen chart. Demographic information, including age, gender, and socioeconomic status, was collected through self-report questionnaires and handled under strict confidentiality procedures. Participation was voluntary and unpaid, and individuals were free to withdraw from the study at any point without penalty. All experimental procedures were non-invasive and approved by the Ethics Committee of the Instituto de Neurociencias, Universidad de Guadalajara (reference ET122023-382). The study adhered to institutional regulations and followed the principles of the Declaration of Helsinki governing research with human participants.

### Apparatus

Stimuli were presented on a 27-inch monitor (1920 × 1080 pixels; refresh rate 60 Hz; mean background luminance ≈ 50 cd/m^2^) in a uniformly lit room. Participants viewed the screen binocularly from 60 to 70 cm, with the head stabilized on a chin rest. The cursor was a small white dot (0.3° of visual angle), driven by a handheld joystick operated with the dominant hand and sampled in synchrony with display refresh. The moving target was a black dot of matched size (0.3°). Trial outcome was signaled by a high-pitched tone (successful interception) or a low-pitched tone (5-s timeout); these auditory cues are the same in all four cohorts (Treviño et al., 2024). The task was implemented in MATLAB using the Psychophysics Toolbox extensions. Sample trial identifiers and per-cohort trial counts are listed in Supplementary Table 1.

### Task and behavior overview

**FIGURE 1:**
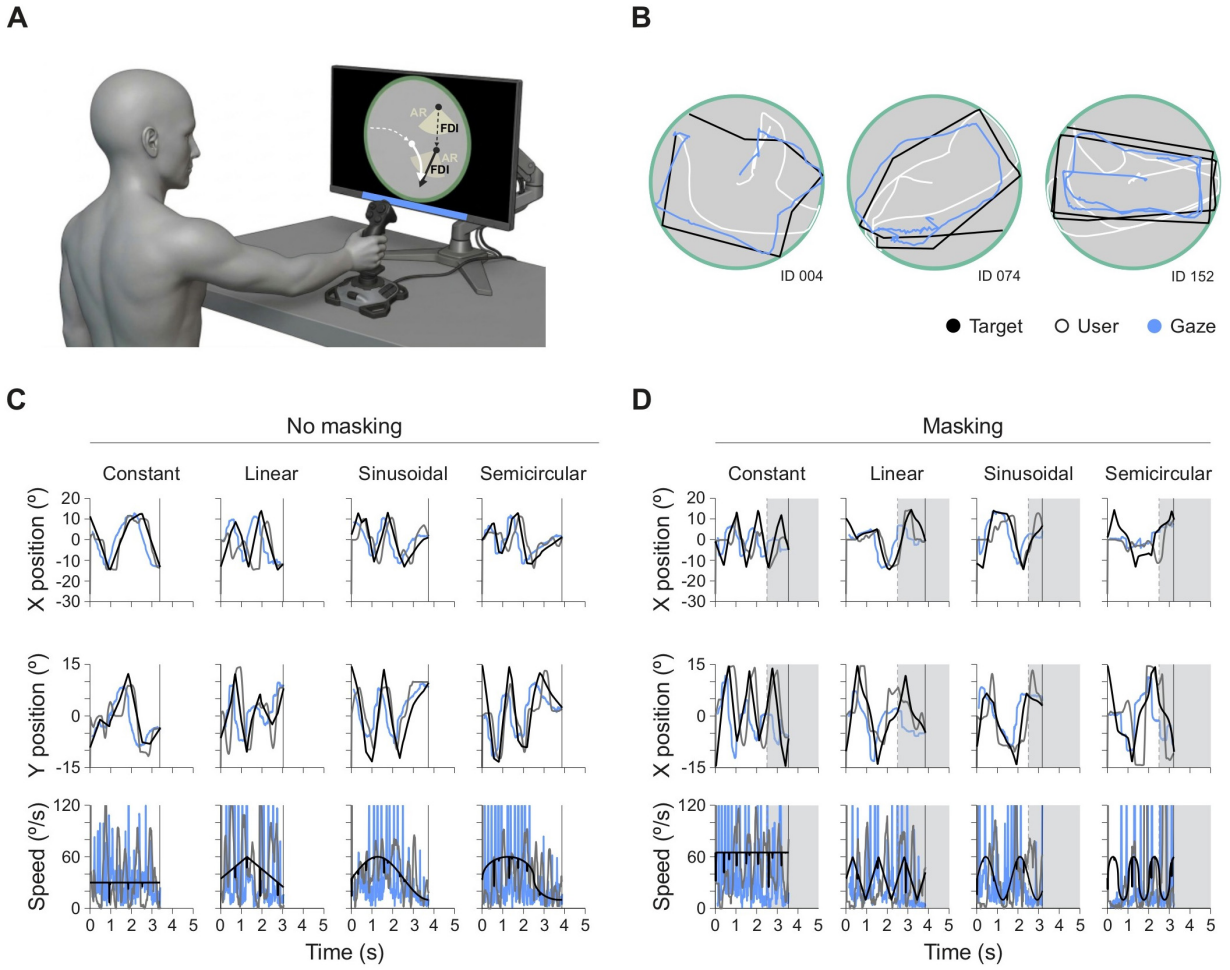
Task and behavioral measures. **(A)** Experimental setup. Participants used a joystick to control a cursor (white) and intercept a moving target (black) displayed on a monitor. The target followed piecewise linear trajectories with periodic direction changes and reflections at arena boundaries. Eye position was continuously recorded with an eye tracker located below the display. **(B)** Representative trajectories from individual trials, showing target (black), cursor (white), and gaze (blue) positions within the arena. Behavioral measures included gaze–target distance (GTD), user–target distance (UTD), and gaze–target and user–target speed differences (Δ*v*_*G*_, and Δ*v*_*U*_). **(C)** Representative trials from the unmasked condition, showing examples of each target speed profile. The top and middle rows depict horizontal and vertical positions of the target (black), cursor (gray), and gaze (blue), respectively, while the bottom row shows instantaneous target (*v*_*T*_), cursor (*v*_*U*_), and gaze (*v*_*G*_) speeds. The vertical solid line indicates target interception. **(D)** Representative trials from the masked condition, organized as in **(C).** The gray shaded region denotes the masking period, beginning at the vertical dashed line (2.5 s), while the vertical solid line indicates target interception. Gaze speed traces appear more variable than target and cursor speed traces because they include both smooth pursuit and rapid catch-up saccades. Trial identifiers for panels **(C)** and **(D)** are provided in Supplementary Table 1.

Participants performed a visuomotor interception task: we asked them to drive a cursor with the joystick to collide with a small moving target while eye position was continuously recorded (Figure 1A). The target moved within a circular arena, following piecewise linear paths with direction changes at fixed-direction intervals (FDIs of 500 ms) that included bounces off the arena boundary; instantaneous *v*_*T*_ followed one of four temporal profiles described below. Each trial began with the user’s cursor at the center of the arena and the target appearing at a random location on the boundary; the trial ended at interception (cursor and target centers within their combined radius, 0.3° + 0.3°; auditory success cue) or at a 5-s timeout (auditory failure cue). Representative trajectories within the arena are shown in Figure 1B; representative position and speed time series from one trial per speed profile are shown in Figure 1C for full-visibility conditions and in Figure 1D for masking conditions.

Participants were instructed to “bring the white cursor closer and collide with the black moving target”. No further instructions were given about how to do so: straight reaches, pursuit-like movements, or hybrid strategies were all possible. Manual behavior was treated as a continuous control signal, we did not segment trials into pursuit and terminal-interception phases in the analyses below; we have used the same continuous framework in a previous paper (Treviño and Márquez, 2025a). Performance was quantified using complementary manual and oculomotor metrics, including speed matching relative to the target and spatial tracking accuracy. Analyses were conducted separately for each speed regime so that the effect of masking could be evaluated within the same kinematic context using within-subject comparisons between full-visibility and masking trials.

Task difficulty was controlled by two parameters previously shown to modulate interception (Treviño et al., 2024; Treviño and Márquez, 2025a): the angular range (AR) of direction changes between FDIs and *v*_*T*_, with larger AR values and higher speeds producing more challenging conditions. When the target reached the arena boundary, its trajectory followed the law of reflection (angle of reflection equal to angle of incidence) with an additional angular variation drawn from the trial-specific AR distribution.

### Experimental design

Four independent cohorts of 40 participants each (160 in total) were tested, one per experiment:

E_1_: Time-varying speed with and without masking (*n* = 40). All three time-varying speed profiles (linear ramps, sinusoidal, semicircular) at five temporal frequencies (0.2–1 Hz), with masked and unmasked trials interleaved 50:50. Masked trials used “tonic” masking: the target became invisible from 2.5 s after trial onset until interception or timeout, regardless of speed-cycle phase.

E_2_: Time-varying speed under acceleration-phase masking at 0° (*n* = 40). Same profiles and frequencies as E_1_; masked trials used phase-locked masking applied during the acceleration phase of each oscillatory cycle (d*v*_*T*_/d*t* > 0; first half of the cycle).

E_3_: Time-varying speed under deceleration-phase masking (*n* = 40). Same profiles and frequencies as E_1_ and E_2_; masked trials used phase-locked masking applied during the deceleration phase of each oscillatory cycle (d*v*_*T*_/d*t* < 0; second half of the cycle).

E_4_: Constant speed (*n* = 40). The constant-*v*_*T*_ condition, with *v*_*T*_ randomly sampled across 13 values from 5 to 65°/s in 5°/s steps. Masked and unmasked trials interleaved 50:50, with tonic masking (no oscillatory cycle is defined in this condition, so phase-locked masking does not apply).

In all cohorts, within-subject manipulations (speed profile, modulation frequency where applicable, phase where applicable, masking condition) were fully permuted and randomly interleaved trial by trial. No block structure was used. Trials in which interception occurred before the masking onset (about 6% of masked trials, see section “Results”) were excluded from masked-window analyses. The total trial count across all four cohorts is 60,000 (see Supplementary Table 1).

Across 60,000 trials pooled over all conditions, 66.0% ended in successful interception. Mean collision times were 2.04 ± 1.18 s (constant, unmasked), 2.28 ± 1.29 s (linear, unmasked), 2.22 ± 1.26 s (sinusoidal, unmasked), and 2.20 ± 1.25 s (semicircular, unmasked). Participants were fast: most successful interceptions occurred before the masking onset at 2.5 s (Supplementary Figure 1; per-cell counts in Supplementary Table 2). On masked trials, 79–82% of successful interceptions terminated before the masking onset and were therefore unaffected by the visual manipulation. The masked-window analyses reported below are based on trials that crossed 2.5 s without intercepting, i.e., the slower, harder portion of the trial distribution. The three masking schemes used across E_1_–E_4_ have been described above. Implementation details, including the phase estimator, are given below.

### Manipulation of target speed (v_*T*_)

The instantaneous *v*_*T*_ was experimentally manipulated to generate different temporal speed profiles during the trial. Speed was defined in degrees of visual angle per second and converted to screen displacement at each frame. Four speed profiles were used to examine how the temporal structure of speed affects interception behavior.

#### Constant speed

In the simplest condition, the target moved with a constant speed (*v*_*C*_) throughout the trial:


$$v_{T}=v_{C}$$

This condition corresponds to classical interception paradigms in which target motion is fully described by a constant velocity vector.

#### Linear piecewise linear oscillations (speed ramps)

In this condition, *v*_*T*_ varied according to piecewise linear functions, generating alternating phases of acceleration and deceleration. This framework unifies both single linear ramps and periodic zigzag (triangular) oscillations within a common formulation. For a single ramp segment, instantaneous speed was defined as:


$$v_{T}\,\left(t\right)=v_{0}+k\,t$$

where *v*_0_ is the initial speed and *k* is the constant rate of change (acceleration or deceleration). This produces a monotonic increase or decrease in speed over time.

To generate oscillatory motion, these linear segments were concatenated into a periodic triangular waveform, such that speed alternated between increasing and decreasing phases. Let *A* denote the amplitude of the oscillation, and *T* its period. The instantaneous speed was defined piecewise within each cycle as:


$$v_{T}\,\left(t\right)=\left\{ \begin{array}{c} v_{m\,i\,n}+\frac{2\,A}{T}\,t_{a\,d\,j},0\leq t_{a\,d\,j}<\frac{T}{2}\\ v_{m\,a\,x}-\frac{2\,A}{T}\,\left(t_{a\,d\,j}-\frac{T}{2}\right),\frac{T}{2}\leq t_{a\,d\,j}<T \end{array}\right.$$

where *t*_*adj*_ = *t* mod *T* represents time within the oscillation cycle, and *v*_*min*_, and *v*_*max*_define the speed bounds. This manipulation preserves a simple temporal structure in which acceleration is constant within each segment, but changes sign abruptly at transition points. Compared to smooth nonlinear profiles (e.g., sinusoidal), this condition introduces discontinuities in acceleration.

#### Sinusoidal speed profile

In this regime *v*_*T*_ varied smoothly according to a sinusoidal function, producing continuous fluctuations between minimum and maximum values:


$$v_{T}\,\left(t\right)=A\cdot s\,i\,n\,\left(2\,\pi \,f_{0}\,t+\phi_{0}\right)+A$$

where *A* determines the amplitude of the oscillation, *f_0_* is the frequency, and ϕ*_0_* defines the initial phase. This profile introduces nonlinear temporal structure while maintaining smooth transitions in speed.

#### Semicircular speed profile

A final profile produced speed variations derived from a semicircular function, such that the magnitude of *v*_*T*_ followed a curved trajectory over time. This generated a nonlinear oscillatory speed profile in which the rate of change in speed varied across the cycle.

We defined *v*_*T*_ for the first half of the oscillation period:


$$v_{T}\,\left(t\right)=\sqrt{A^{2}-{\left(\frac{2\,A}{T/2}\,t_{a\,d\,j}-A\right)}^{2}+A}$$

and for the second half:


$$v_{T}\,\left(t\right)=-\sqrt{A^{2}-{\left(\frac{2\,A}{T/2}\,t_{a\,d\,j}-A\right)}^{2}+A}$$

Together, these profiles span a hierarchy of kinematic complexity: constant speed provides a baseline with no temporal variation; linear ramps introduce constant acceleration; and the two oscillatory profiles introduce time-varying acceleration. Sinusoidal and semicircular forms were chosen to differ in the higher-order structure of acceleration, so comparing them at matched *v*_*T*_ range isolates the contribution of acceleration structure from that of speed range.

### Visual masking

To examine interception behavior in the absence of continuous visual feedback, a subset of trials included target masking, in which the moving target became invisible after an initial observation period while continuing its trajectory. Masking was implemented by setting target contrast to 0%, rendering it visually undetectable while its motion continued internally within the program. Masked and unmasked trials were intermixed in equal proportions (50:50), randomly permuted within each session. Half of the trials therefore proceeded under full visibility throughout, and half had the target rendered invisible from 2.5 s after trial onset until interception or trial timeout. Three masking schemes were used across cohorts: (1) tonic masking (E_1_, E_4_), in which disappearance occurred regardless of motion phase; (2) acceleration-phase masking (E_2_), applied during the acceleration phase of the oscillatory cycle (d*v*_*T*_/d*t* > 0); and (3) deceleration-phase masking (E_3_), applied during the deceleration phase (d*v*_*T*_/d*t* < 0).

The two phase-specific schemes tested whether the cost of brief visual loss depends on when in the oscillatory cycle it occurs, and therefore on the sign of d*v*_*T*_/d*t* at that moment. The phase of the oscillation at time *t* was estimated relative to the oscillation period and initial phase offset, allowing masking to occur at controlled time points within the speed cycle.


$$t_{\mathrm{percentile}}=\frac{\mathrm{mod}\,\left(t+\phi_{\mathrm{lag}},T\right)}{T}$$

where *T* represents the oscillation period and ϕ_*lag*_ accounts for the initial phase offset. During masked intervals, participants continued to control the joystick and attempted to intercept the invisible target based on internal predictions of its motion. By combining controlled manipulations of *v*_*T*_ with the transient removal of visual feedback, this design isolated how the temporal structure of *v*_*T*_ constrains predictive visuomotor interception.

Behavioral metrics used for comparisons were computed exclusively within the 2.5–5 s window. This interval allowed a direct comparison between masked and unmasked conditions because, during these 2.5 s, masked trials had no visible target, while unmasked trials still did; any difference within this time window therefore isolates the contribution of continuous visual feedback while controlling for trial onset, motor preparation, and overall task structure.

### Eye tracking

Binocular eye movements were recorded using the Tobii Pro Fusion eye tracker (Tobii Pro SDK for Windows; Stockholm, Sweden) at a sampling rate of 60 Hz (Treviño and Márquez, 2025a). Head stabilization on the chin rest used in the apparatus also minimized gaze drift (Márquez et al., 2024; Treviño and Márquez, 2025a). Calibration routines were performed at the beginning of each session to assess tracking accuracy. These routines included a standard four-point spatial calibration, followed by a smooth-pursuit validation procedure in which participants tracked a slowly moving dot (10°/s) to estimate gaze gain. Additionally, alternating black–white screen cycles were used to evoke the pupillary light reflex and verify signal integrity (Treviño and Márquez, 2025a). Participants were instructed to move their eyes naturally during the task and were not given explicit instructions regarding gaze movements (Márquez and Treviño, 2024b; Treviño and Márquez, 2025a; Harris et al., 2024). This approach allowed natural and spontaneous gaze behavior to occur during visuomotor interception.

In this study, we did not classify eye movements into fixations, saccades, or smooth pursuit. Instead, our analyses used continuous gaze-position and gaze-velocity signals computed at each recorded frame. Two practical reasons motivated this choice. First, the interception task involved dynamic, continuous tracking of a moving target, in which different oculomotor behaviors often overlap in time and may occur within very short intervals; under those conditions, strict event classification requires dynamic, context-dependent thresholds and can introduce segmentation errors at moderate sampling rates (Andersson et al., 2017). Second, at 60 Hz, fast saccade kinematics cannot be resolved, so changes in gaze velocity could, in principle, reflect altered saccade frequency or amplitude rather than altered tracking gain. Continuous gaze metrics have been used in studies of visuomotor tracking and interception precisely because they allow direct comparison between gaze velocity and target velocity, and avoid biases associated with event-detection algorithms (Holmqvist et al., 2011; Márquez and Treviño, 2024b; Spering and Montagnini, 2011; Treviño et al., 2026).

Although event-classification approaches have been applied with similar hardware in interception contexts (e.g., (Treviño and Márquez, 2025b), our continuous-signal framework avoids the threshold-dependent segmentation errors that could arise at 60 Hz. Nevertheless, as a sensitivity check for the present analyses, we re-computed *v*_*G*_ and GTD after excluding frames with instantaneous gaze velocity above 100°/s (a conservative saccade-exclusion threshold). The qualitative pattern was preserved across all conditions, and GTD remained essentially unchanged at the participant level (Pearson *r* > 0.99 relative to the original; Supplementary Figure 2 and Supplementary Table 3).

### Pupillometry

The Tobii Pro Fusion provides per-eye pupil diameter (in mm) sampled at 60 Hz; an equivalent pupil-area estimate is also available in the SDK. We averaged the pupil-diameter signals across the two eyes and treated the resulting continuous trace as a single signal. After blink removal and linear interpolation of short gaps, the mean of the first 30 frames (∼500 ms) was subtracted per trial as a baseline. Two temporal windows were extracted: an early window (frames 131–150, ∼333 ms before masking onset) and a late window (frames 281–300, aligned with the end of the trial). For each speed profile, a 2 × 2 repeated-measures ANOVA was applied with factors window (early vs late) and visibility (unmasked vs masked), followed by Bonferroni-corrected pairwise contrasts. This analysis was exploratory: we did not pre-specify a hypothesis about pupil sensitivity to motion structure. We used the slow timescale of the pupil response (∼1 s) to ask whether it tracked integrated task variables rather than instantaneous motion (Márquez and Treviño, 2024b; Mathôt and Vilotijević, 2023).

A potential confound could be the small change in mean screen luminance when the 0.3° target vanishes. Geometric considerations make this contribution small: the target subtends ∼4.5 × 10^−5^ of the visible screen area, corresponding to a mean-luminance change of ∼0.002 cd/m^2^ against a 50 cd/m^2^ gray background. However, to test this potential contribution empirically, we split late-window frames by whether the gaze was within 4° of the (still-tracked) target position (“near”) or farther (“far”). If local luminance at the gaze position drove the pupil reduction, the masked-near cell should show a larger reduction than the masked-far. A 2 × 2 (visibility × gazelocus) repeated-measures ANOVA on late-window pupil size directly tested this.

## Data processing and analysis

Gaze and joystick signals were imported into MATLAB (R2025b) for offline analysis. Preprocessing included removal of blink and signal artifacts, followed by linear interpolation to reconstruct short segments of missing data (Fink et al., 2024; Holmqvist et al., 2011; Mathôt and Vilotijević, 2023; Treviño and Márquez, 2025a). All spatial coordinates were converted to degrees of visual angle prior to further analysis.

We used several metrics to quantify gaze behavior, manual control, and visuomotor coordination: Gaze speed (*v*_*G*_): instantaneous gaze velocity (°/s), derived from the first derivative of gaze position. Characterizes visual tracking dynamics and allows direct comparison with target motion (Treviño and Márquez, 2025a); User speed (*v*_*U*_): Instantaneous velocity (°/s) of the participant-controlled cursor. Reflects the motor response used to pursue and intercept the target (Treviño et al., 2024; Treviño and Márquez, 2025a); Gaze–target distance (GTD): Euclidean distance (°) between gaze and target. Smaller values indicate more precise visual tracking; Gaze–user distance (GUD). Euclidean distance (°) from gaze to the participant-controlled dot; offers insight into self-monitoring and eye–hand coordination (Treviño and Márquez, 2025a); User–target distance (UTD). Euclidean distance (°) between the participant-controlled cursor and the target. Captures spatial proximity to interception; Speed-matching metrics. Δ*v*_*G*_ = *v*_*G*_ − *v*_*T*_; Δ*v*_*U*_ = *v*_*U*_ − *v*_*T*_. Values near zero indicate accurate speed matching; positive or negative deviations reflect over- or undershoot of the target’s instantaneous speed.

### Statistical analysis

We used a preliminary study with ten participants to estimate the expected magnitude of gaze modulation during rapid *v*_*T*_ changes. In this pilot, *v*_*T*_ varied sinusoidally between 10°/s and 60°/s at 0.2 Hz, and *v*_*G*_ was compared between the minimum and maximum phases of the cycle. A paired-sample *t*-test revealed a large effect size (Cohen’s *d* = 1.44). A subsequent power analysis, assuming α = 0.05 and statistical power = 0.95, indicated that nine participants would be sufficient to detect reliable modulation of *v*_*G*_ by *v*_*T*_. The sample sizes used in the present experiments exceeded this requirement.

For within-subject comparisons, parametric tests were applied when statistical assumptions were satisfied. Within-subject effects were assessed using repeated-measures ANOVA (RM-ANOVA). When parametric assumptions were violated or when ordinal comparisons were required, the Friedman test (repeated-measures designs) and the Kruskal–Wallis test (independent groups) were used (Treviño and Márquez, 2025a). For comparisons across conditions in the same participants, paired *t*-tests at specific speed or frequency levels and RM-ANOVA models with factors such as Condition and *v*_*T*_ (or Frequency) were used. For independent-group comparisons, independent-samples *t*-tests and mixed-design ANOVAs were used.

Relationships between target parameters (e.g., speed or oscillation frequency) and behavioral measures were quantified using linear and quadratic regression models, with overall model significance evaluated using ANOVA. For quadratic fits, overall model significance was tested with the *F*-statistic of the full model; the contribution of the quadratic term was tested using a partial *F*-test comparing the full model with a linear-only nested model. Both statistics are reported whenever a quadratic fit is presented. Zero-crossing analyses were applied to the speed-difference metrics (Δ*v*_*G*_, Δ*v*_*U*_) by estimating the *v*_*T*_ value at which the mean curve crossed zero using linear interpolation; statistical significance of the crossings was assessed using one-sample *t*-tests against zero.

Effect sizes were reported as Cohen’s *d* for *t*-tests and partial *η^2^* for ANOVA models. When appropriate, Hedges’ g was also reported to correct for small-sample bias. Group-level results are reported as mean ± SEM. We refer to the overall *F*-test of an ANOVA (the test that asks whether any condition mean differs from the others) as the “omnibus test.” Pairwise contrasts following an RM-ANOVA were Bonferroni-corrected within each metric. Pairwise comparisons reported in the absence of a significant omnibus effect are explicitly labelled exploratory. Statistical significance was defined as *p* ≤ 0.05.

Statistical comparison of the four v_*T*_ models (Constant, Linear, Sine, Semicircular) was performed by analysing Root Mean Square Error (RMSE) across four dependent variables: Δv_*G*_, GTD, Δv_*U*_ and UTD. As a general evaluation of model efficacy, an Overall Performance Score was derived for each group: for every dependent variable, groups were ranked from 1 (lowest RMSE) to 4 (highest RMSE), and the mean rank across variables was transformed into a standardized performance metric using the inverted scale (5 − mean_rank) × 100, with higher scores indicating superior tracking performance.

Above-chance performance in this study was defined relative to a yoked-control random-policy benchmark. The idea is to ask whether observed interception rates exceed what would be obtained if the cursor trajectory bore no causal relationship to the target trajectory. For each participant, the real cursor trajectories were paired with target trajectories drawn from other trials of the same (profile, visibility) cell, and the scoring procedure was replayed exactly (cursor and target within 0.59°, the empirical 95th percentile of inter-object distance at successful interception). 500 such yokings per cell produced a null distribution of interception rates. Therefore, “above-chance” refers to real rates exceeding the 95th percentile of this null. All 320 participant × cell combinations exceeded their own 95th percentile (see Supplementary Figure 3).

## Results

### Interception behavior under constant target speed

**FIGURE 2:**
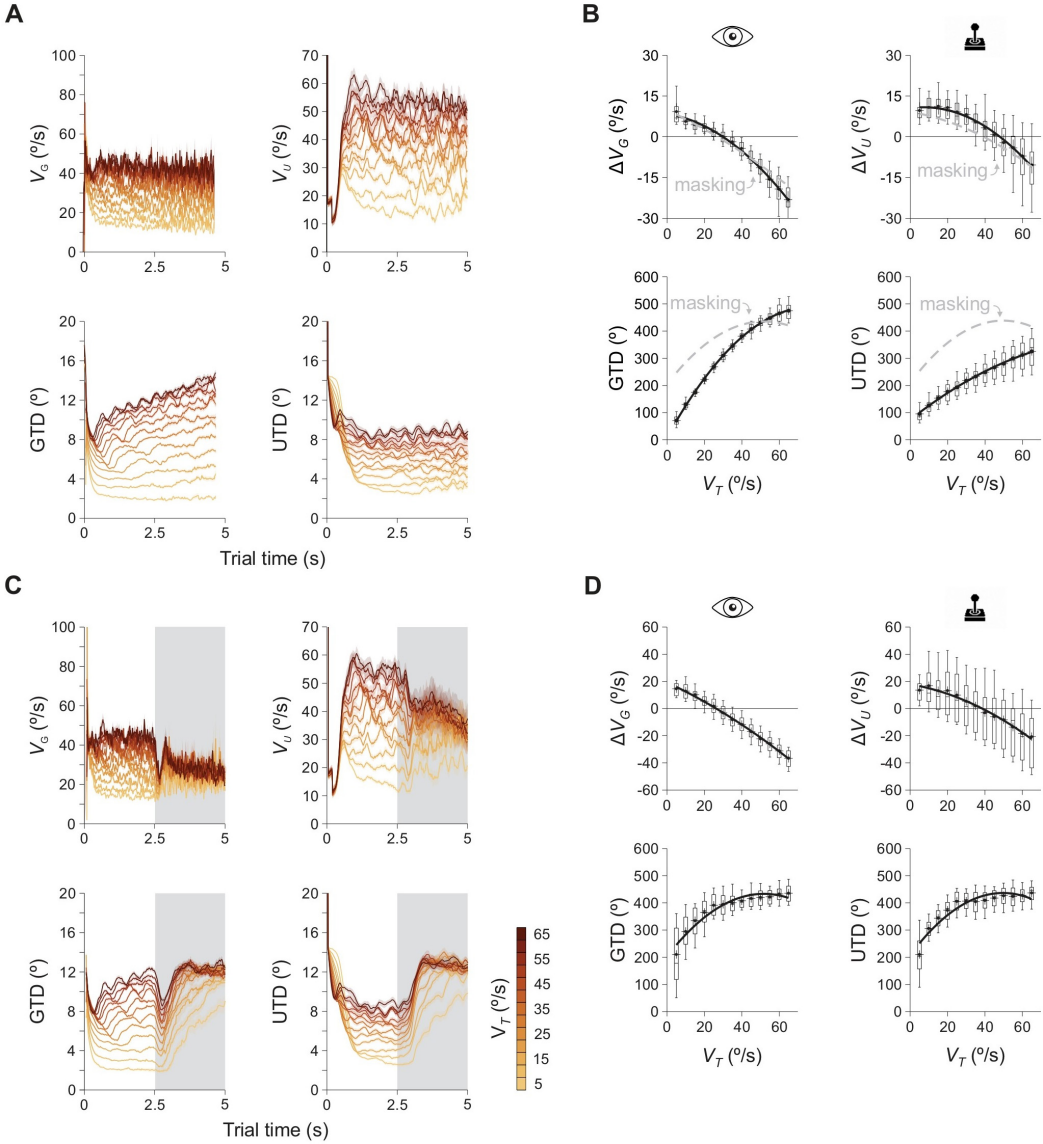
Baseline interception under constant target speed. **(A)** Group-average temporal traces (0–5 s) of gaze speed (*v*_*G*_), GTD, user speed (*v*_*U*_), and UTD across constant target speeds (*v*_*T*_ = 5–65°/s). Shaded regions in temporal traces denote SEM. **(B)** Summary metrics computed from the second half of the trial (2.5–5 s): Δ*v*_*G*_, GTD, Δ*v*_*U*_, and UTD as functions of *v*_*T*_ (whisker plots reflect participant averages). Gray dotted lines indicate average fits from the masked condition for direct comparison. **(C)** Temporal traces under masking (gray patch, 2.5–5 s), during which the target was not visible. **(D)** Summary metrics under masking. Eye and joystick icons are included to facilitate identification of gaze- and manual-control measures, respectively. The joystick icon serves only as a visual cue for manual control and does not represent the specific joystick used for the experiments (illustrated in Figure 1A), which provided continuous analog input.

We first examined interception behavior under constant *v*_*T*_ (*a* = 0), which served as a baseline for visuomotor control. In this regime, participants could rely on a stable estimate of target velocity to guide both gaze and manual movements. *v*_*T*_ values were randomly sampled across 13 levels ranging from 5 to 65°/s (Figure 2A). Data are based on 40 participants.

We turned first to oculomotor behavior, using Δ*v*_*G*_ (Δ*v_*G*_* = *v*_*G*_ − *v*_*T*_) as a function of *v*_*T*_. Gaze speed depended strongly on *v*_*T*_ (repeated-measures ANOVA: *F*_(12,468)_ = 374.71, *p* < 0.001, partial *η^2^* = 0.91). *v*_*G*_ tended to move faster than the target at lower *v*_*T*_ and slower at higher *v*_*T*_. The transition between these regimes occurred at approximately 26.20°/s (*p* < 0.05; upper left panel in Figure 2B). We quantified spatial gaze accuracy using the gaze–target distance (GTD). GTD increased with *v*_*T*_, indicating reduced spatial precision at higher speeds (repeated-measures ANOVA: *F*_(12,468)_ = 356.19, *p* < 0.001, partial *η^2^* = 0.90; lower left panel in Figure 2B).

Manual speed matching was quantified using Δ*v*_*U*_ (Δ*v_*U*_* = *v*_*U*_ − *v*_*T*_) as a function of *v*_*T*_, where positive values indicate movements faster than the target and negative values indicate slower movements. A repeated-measures ANOVA revealed a strong effect of *v*_*T*_ (*F*_(12,468)_ = 61.35, *p* < 0.001, partial *η^2^* = 0.61), indicating systematic modulation of manual speed across conditions. Participants tended to overshoot the target at lower speeds and progressively lag behind it at higher speeds. A zero-crossing analysis using 95% confidence intervals identified a transition point at approximately 42.27°/s (*p* < 0.05), marking the shift between these regimes (upper right panel in Figure 2B). Notably, the transition point for gaze (∼26 °/s) was lower than for manual responses (∼42 °/s), indicating that gaze tracking shifts from overshooting to undershooting at a lower *v*_*T*_ than manual control. To assess spatial interception performance, we analyzed the user–target distance (UTD) as a function of *v*_*T*_, with smaller values indicating more accurate interception. UTD increased monotonically with *v*_*T*_, indicating that spatial errors increased as targets moved faster. A repeated-measures ANOVA confirmed a strong effect of *v*_*T*_ on UTD (*F*_(12,468)_ = 356.19, *p* < 0.001, partial *η^2^* = 0.90; lower right panel in Figure 2B).

In masking trials, the target disappeared between 2.5 and 5 s after trial onset while the participant’s cursor remained visible (Figure 2C). Behavioral metrics were extracted from the 2.5–5 s interval (see section “Materials and methods”). A transient dip in v_*G*_ and GTD is visible at the masking onset (∼2.5 s); this reflects a brief stabilization of gaze when the target vanishes: pursuit velocity drops sharply, and GTD transiently improves because gaze lingers near the last seen target position before drift accumulates over the subsequent ∼500 ms. The v_*G*_–v_*T*_ dependence persisted under masking, but absolute Δv_*G*_ grew (t_(518)_ = 10.35, *p* < 0.001, *d* = 0.45; 11/13 speeds; upper left, Figure 2D). Speed-specific comparisons revealed pervasive masking effects on the gaze-based metrics: 12 of 13 speeds were FDR-significant for Δv_*G*_ (only 25°/s did not survive correction) and 11 of 13 for GTD (45°/s and 50°/s did not survive). UTD was modulated by masking at every speed tested (13/13). Manual speed error (Δv_*U*_) was less sensitive, only 4 of 13 speeds reached FDR significance, all in the higher range (45, 55, 60, 65°/s). The dissociation parallels the pattern reported in time-varying conditions: masking impacts gaze-based tracking and spatial error more strongly than manual speed control. Full per-speed statistics are shown in Supplementary Table 4, and the matching per-speed bar plots are provided in Supplementary Figure 4. GTD followed a similar pattern (*r* = 0.67 across conditions; t_(519)_ = −54.72, *p* < 0.001, *d* = −2.40; 11/13 speeds; lower left, Figure 2D).

Δ*v*_*U*_ retained its dependence on *v*_*T*_ under masking but showed a modest upward shift (*r* = 0.63; *t*_(519)_ = 3.72, *p* < 0.001, *d* = 0.16; 6/13 speeds; upper right, Figure 2D). UTD was the most affected metric: structure preserved, magnitude markedly increased at every speed (*r* = 0.67; *t*_(519)_ = −54.72, *p* < 0.001, *d* = −2.40; 13/13; lower right, Figure 2D). Together, these results indicate that, under constant *v*_*T*_, both gaze and manual interception retain their overall speed-dependent structure when visual feedback is removed, but performance degrades substantially, particularly in spatial accuracy.

### Target speed changing with constant acceleration (linear speed ramps)

**FIGURE 3:**
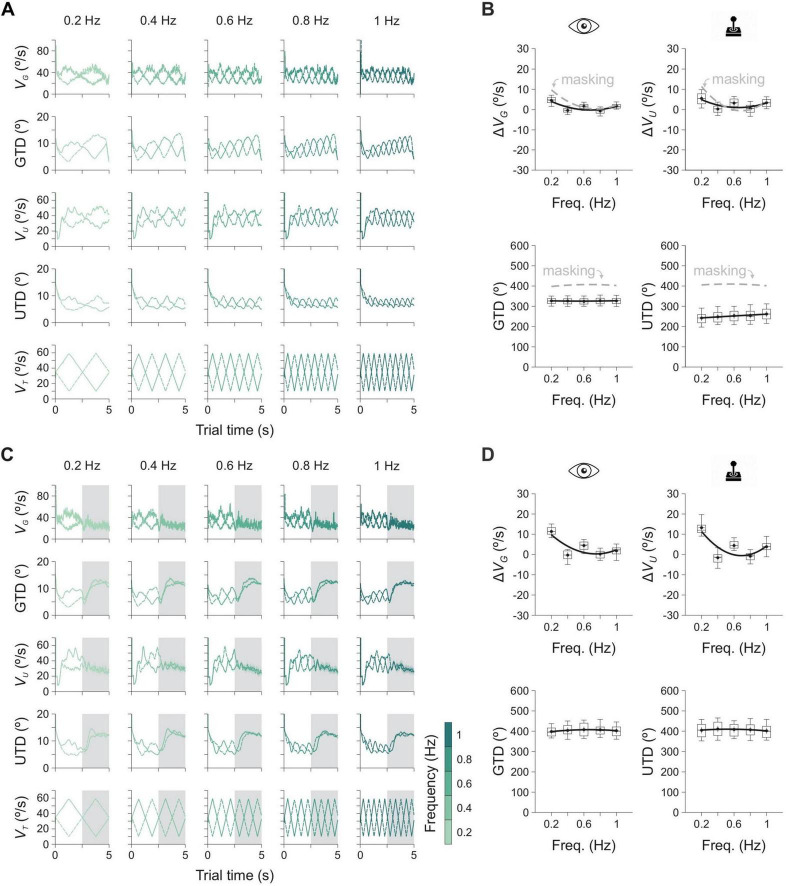
Interception under linear speed ramps. **(A)** Group-average traces of gaze and manual behavior during periodic linear changes in *v*_*T*_ across temporal frequencies (left to right). Opposing phases (0°/180°) were counterbalanced. Bottom row shows imposed *v*_*T*_ profiles. **(B)** Summary metrics (2.5–5 s) as functions of ramp frequency: Δ*v*_*G*_, GTD, Δ*v*_*U*_, and UTD. Lines indicate best-fitting models (linear or quadratic). Overlaid gray dotted lines represent fits from masking conditions for comparison. **(C)** Same analyses under masking (gray patch indicates occlusion window). **(D)** Summary metrics under masking illustrating deviations induced by loss of visual input.

We next examined interception performance under linearly varying *v*_*T*_, in which velocity changed over time according to constant-acceleration and constant-deceleration profiles. In this condition, *v*_*T*_ followed periodic linear ramps, increasing and decreasing within each cycle. The rate of change was controlled by the temporal frequency of the ramps (0.2, 0.4, 0.6, 0.8, and 1 Hz), corresponding to progressively faster speed variations (Figure 3A). As in the constant-speed condition, masking was applied in half of the trials by removing visual feedback after an initial observation period while the cursor remained visible. All behavioral metrics used for comparisons were averaged within the masked interval, and ramp frequency was treated as the primary independent variable. All conditions (frequency, phase, and masking) were randomly permuted within participants.

Across the four metrics, three were insensitive to ramp frequency under full visibility: Δv_*G*_ (*F*_(4,476)_ = 0.71, *p* = 0.59, partial η^2^ = 0.006; upper left, Figure 3B), GTD (*F*_(4,476)_ = 0.73, *p* = 0.57, partial η^2^ = 0.006; lower left), and Δv_*U*_ (*F*_(4,476)_ = 1.41, *p* = 0.23, partial η^2^ = 0.01; upper right). None of the three showed a zero-crossing across frequencies, in contrast to the over- to under-shoot transition seen across v_*T*_ in the constant-speed condition. UTD was the exception: spatial error grew with ramp frequency (*F*_(4,476)_ = 22.58, *P* < 0.001, partial η^2^ = 0.16; lower right), again with no zero-crossing.

We illustrate the masking effects in Figure 3C: group-averaged temporal traces with gray patches marking the occlusion window. These overlays allow direct visualization of how the time course of each dependent variable changed following the removal of visual input. Masking widened the gaze metrics at every frequency: Δ*v*_*G*_ grew (*t*_(798)_ = −17.68, *p* < 0.001, *d* = −1.44; upper left, Figure 3D) and GTD grew substantially more (*t*_(798)_ = −37.79, *p* < 0.001, *d* = −3.09; lower left). Manual speed matching was preserved overall: Δ*v*_*U*_ differed at 3 of 5 frequencies but showed no pooled effect (*t*_(798)_ = −1.53, *p* = 0.13, *d* = −0.13; upper right). UTD, in contrast, was the most affected (5/5 frequencies; *t*_(798)_ = −52.46, *p* < 0.001, *d* = −4.28; lower right), a similar gaze-vs-hand dissociation seen under constant *v*_*T*_.

### Acceleration and deceleration at matched instantaneous velocity

The linear-ramp condition provides a direct test of whether predictive control reflects higher-order kinematic information beyond instantaneous velocity. Within each cycle, v_*T*_ adopts the same value twice (per cycle): once during the acceleration phase (*k* > 0), and once during the deceleration phase (*k* < 0). We reasoned that a representation relying solely on local velocity should produce indistinguishable behavior at matched v_*T*_. However, a system sensitive to the sign of acceleration, or any other higher-order signal, should not.

For each frame we identified the cycle phase from the sign of d*v*_*T*_/dt, binned *v*_*T*_ into 5°/s intervals from 10 to 60°/s, and computed per-participant means of Δ*v*_*G*_, GTD, Δ*v*_*U*_, and UTD separately for the acceleration and deceleration phases. Paired contrasts at matched *v*_*T*_ revealed reliable differences between phases across all four metrics and all velocity bins (10/10 bins FDR-significant for each metric; Supplementary Figure 5 and Supplementary Table 5). Behavior at the same instantaneous velocity differed systematically depending on whether the target was accelerating or decelerating.

This rules out a strict local-velocity account: the visuomotor signal contains additional kinematic information beyond instantaneous speed, most parsimoniously, the sign of acceleration, and that information shapes the predictive response. The result is consistent with neurophysiological evidence that MT and MST neurons can carry acceleration-related signals in smooth-pursuit contexts (Lisberger and Movshon, 1999), and with reaching studies in which manual responses differ between the acceleration and deceleration phases of target motion (de Brouwer et al., 2002).

### Interception behavior under non-linear speed profiles

**FIGURE 4:**
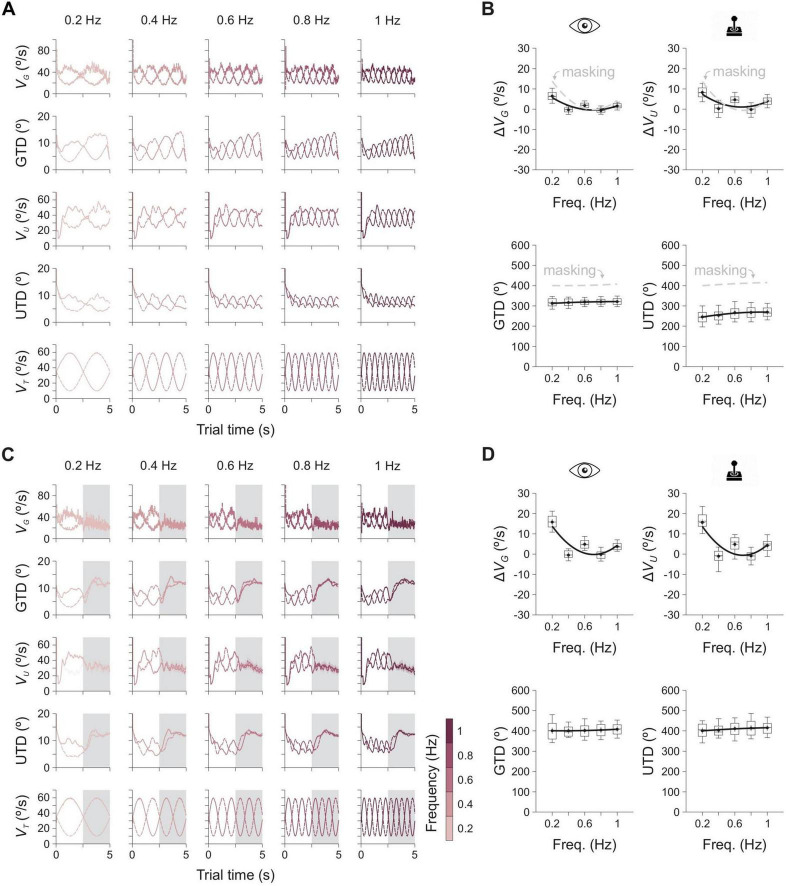
Interception under sinusoidal speed modulation. **(A)** Group-average traces of gaze and manual behavior during sinusoidal changes in target speed (*v*_*T*_) across temporal frequencies (left to right). Bottom row shows the imposed sinusoidal *v*_*T*_ profiles. **(B)** Summary metrics computed during the analysis window (2.5–5 s): Δ*v*_*G*_, GTD, Δ*v*_*U*_, and UTD as functions of modulation frequency. Lines indicate best-fitting models (linear or quadratic). Overlaid gray dotted lines represent fits obtained under masking conditions for comparison. **(C)** Same analyses as in **(A)** under masking, with the gray patch indicating the target occlusion window. **(D)** Summary metrics under masking illustrating the effects of visual occlusion on tracking and interception performance. Corresponding analyses for semicircular speed modulation are shown in Supplementary Figure 6.

We next examined interception behavior under nonlinear *v*_*T*_ profiles, in which *v*_*T*_ varied continuously according to sinusoidal or semicircular functions. These trajectories impose time-varying acceleration, so a constant-speed or linear-ramp approximation can no longer apply continuously across the trial. Masking, frequencies (0.2–1 Hz), and analysis window were the same as in the linear-ramp condition (see section “Materials and methods”). Because sinusoidal (Figure 4) and semicircular (Supplementary Figure 6) trajectories produced highly similar statistical patterns, their results are described in parallel.

Δv_*G*_ was insensitive to modulation frequency in both profiles (sinusoidal: *F*_(4,476)_ = 1.59, *p* = 0.18, partial η^2^ = 0.013; semicircular: *F*_(4,476)_ = 1.89, *p* = 0.11) but increased under masking at every frequency (sinusoidal: t_(798)_ = −19.06, *p* < 0.001, Cohen’s *d* = −1.56; semicircular: t_(798)_ = −19.00, *p* < 0.001, *d* = −1.55). GTD grew with frequency in both profiles (sinusoidal: *F*_(4,476)_ = 4.01, *p* = 0.003, partial η^2^ = 0.033; semicircular: *F*_(4,476)_ = 7.23, *p* < 0.001, partial η^2^ = 0.057) and grew at every frequency under masking (sinusoidal: t_(798)_ = −36.03, *p* < 0.001, *d* = −2.94; semicircular: t_(798)_ = −42.23, *p* < 0.001, *d* = −3.44).

Δ*v*_*U*_ showed a frequency effect under sinusoidal modulation (*F*_(4,476)_ = 4.94, *P* < 0.001, partial *η^2^* = 0.040; no zero-crossing), but not under semicircular (*F*_(4,476)_ = 2.24, *p* = 0.06, partial *η^2^* = 0.018; upper right). Masking increased Δ*v*_*U*_ in both (sinusoidal: 3/5 frequencies, *t*_(798)_ = −2.65, *p* = 0.008, *d* = −0.22; semicircular: 5/5, *t*_(798)_ = −5.13, *p* < 0.001, *d* = −0.41). UTD increased with frequency in both profiles (sinusoidal: *F*_(4,476)_ = 43.46, *p* < 0.001, partial *η^2^* = 0.27; semicircular: *F*_(4,476)_ = 46.09, *p* < 0.001, partial *η^2^* = 0.28) and worsened at every frequency under masking (sinusoidal: *t*_(798)_ = −48.27, *p* < 0.001, *d* = −3.94; semicircular: *t*_(798)_ = −45.29, *p* < 0.001, *d* = −3.69).

### No strategic reallocation of gaze under masking

One concern is that the masking-induced rise in GTD could reflect a strategic shift in where participants look (e.g., toward the cursor) rather than a failure of predictive tracking. We tested this idea directly on the 100 frames following masking onset by quantifying the fraction of gaze samples within 2° of the cursor and within 2° of the (still-tracked) target position, plus the spatial standard deviation of gaze around the expected target path.

Cursor-fixation fraction increased under masking, from 4 to 6% in unmasked trials to 17–20% in masked trials, a small but consistent shift (Constant: dz = 0.89; Linear: dz = 0.94; Sinusoidal: dz = 0.93; Semicircular: dz = 0.95; all p_*Bonf*_ < 10^−5^). Even under masking, however, the cursor-fixation fraction remained low (well below 20%), indicating that participants did not adopt cursor-monitoring as a predominant strategy under masking. The complementary “neither” fraction (gaze close to neither the cursor nor the predicted target) dropped sharply under masking (from ∼92% to ∼78%, all dz around 1.0; p_*Bonf*_ < 10^−6^), showing that gaze re-organized around both objects when the target disappeared (Supplementary Figure 7).

Gaze spatial dispersion around the expected target path increased dramatically under masking, and the effect was *v*_*T*_ profile-dependent (profile × visibility: *F*_(2,78)_ = 3.31, *p* = 0.042). The increase was small for constant motion (9.4°→ 9.8°, *dz* = 0.44) and large for the three time-varying profiles (Linear: 7.9°→ 9.6°, *dz* = 2.4; Sinusoidal: 8.1°→ 9.6°, *dz* = 1.9; Semicircular: 8.3°→ 9.7°, *dz* = 1.8; all *p*_*Bonf*_ < 10^–2^). Therefore, participants remained target-oriented, but the spatial precision of that target-oriented behavior degraded most when the local-velocity approximation broke down.

Two-dimensional gaze density maps centered on the expected target position and aligned to its motion direction (Supplementary Figure 7) showed a consistent across-motion bias of approximately −3° in the masked condition across all profiles, reflecting the typical position of the cursor below the target during the closing maneuver. The “strategic” component is therefore small and uniform, while the predictive component (gaze dispersion) is v_*T*_ profile-dependent. The GTD increase under masking is not explained by a strategic reallocation of gaze toward the cursor or toward any single locus away from the expected target path.

### Comparison across temporal speed profiles: Linear and nonlinear

On unmasked trials, we compared the three time-varying profiles within subjects (frequencies and metrics as in the previous sections). Δ*v*_*G*_ showed a small profile effect (*F*_(2,238)_ = 4.07, *p* = 0.018, partial *η^2^* = 0.033) but no frequency effect or interaction (both *p* > 0.28), and was largely stable across conditions (first row, Supplementary Figure 8). GTD depended strongly on profile (*F*_(2,238)_ = 86.86, *p* < 0.001, partial *η^2^* = 0.422), on frequency (*F*_(4,476)_ = 7.08, *p* < 0.001, partial *η^2^* = 0.056), and on their interaction (*F*_(4,476)_ = 3.27, *p* = 0.012, partial *η^2^* = 0.027; second row, Supplementary Figure 8). Δ*v*_*U*_ was insensitive to profile (*F*_(2,238)_ = 2.13, *p* = 0.121, partial *η^2^* = 0.018) and to the profile × frequency interaction (*F*_(4,476)_ = 1.30, *p* = 0.268), but increased with frequency (*F*_(4,476)_ = 5.58, *p* < 0.001, partial *η^2^* = 0.045; third row, Supplementary Figure 8). UTD was the most strongly modulated: profile (*F*_(2,238)_ = 137.17, *p* < 0.001, partial *η^2^* = 0.536), frequency (*F*_(4,476)_ = 98.75, *p* < 0.001, partial *η^2^* = 0.454), and their interaction (*F*_(4,476)_ = 3.95, *p* = 0.0036, partial *η^2^* = 0.032; fourth row, Supplementary Figure 8). Masked-trial comparisons across profiles are reported in the next subsection.

### Differential impact of target masking across motion models

**FIGURE 5:**
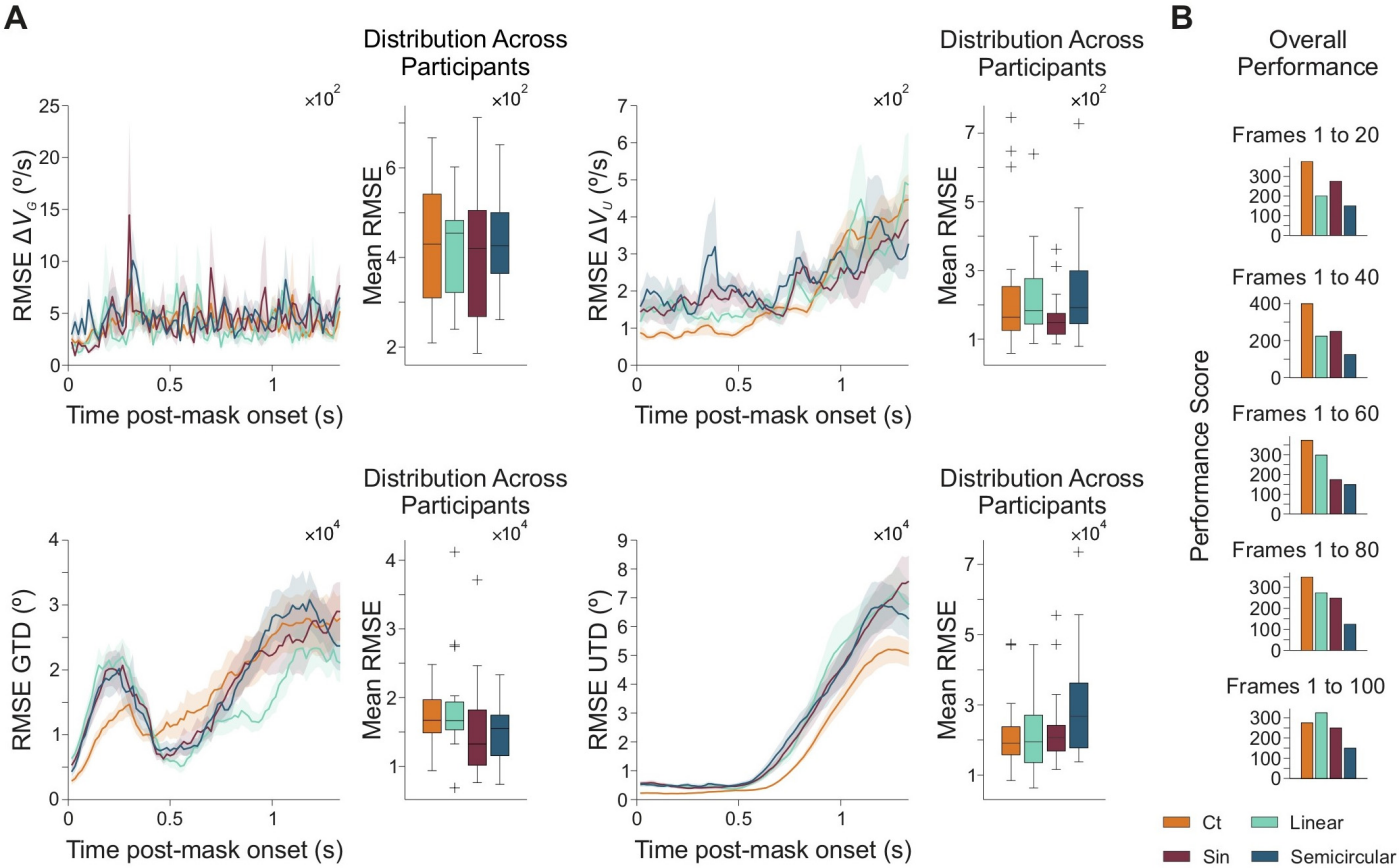
Temporal emergence of masking-induced errors. Time-resolved effects of masking quantified as RMSE between masked and unmasked conditions. **(A)** Evolution of RMSE for Δ*v*_*G*_, GTD, Δ*v*_*U*_, and UTD aligned to masking onset. Constant-*v*_*T*_ profile is shown in orange, linear profile in teal, sinusoidal profile in pink, and semicircular profile in blue. **(B)** Cumulative performance indices computed over progressively increasing time windows following masking onset. This analysis illustrates how performance diverges across motion models over time, with constant and linear profiles showing delayed error accumulation relative to nonlinear dynamics. Same color code for all panels.

To determine how the temporal structure of *v*_*T*_ constrains predictive control under visual occlusion, we compared performance across the four speed profiles. Because speed-difference metrics (Δ*v*_*G*_ and Δ*v*_*U*_) contain signed deviations, performance loss was quantified using root mean square error (RMSE), which captures error magnitude independently of direction (Figure 5A; group average traces in Supplementary Figure 9).

Root mean square error (RMSE) was computed for Δ*v*_*G*_, GTD, Δ*v*_*U*_, and UTD as the difference between masked and unmasked conditions during the occlusion window (2.5–5 s), allowing performance to be evaluated relative to each participant’s own visible baseline. This within-subject comparison isolates the contribution of continuous visual input while controlling for intrinsic task difficulty and inter-individual variability. Consistent with prior occlusion paradigms (Adams et al., 2012; Kreyenmeier et al., 2021, 2022), performance loss was defined relative to a visible baseline. Under this framework, smaller RMSE values indicate better preservation of performance during occlusion. Analyses were restricted to a 100-frame window following masking onset and averaged across modulation frequencies to capture the immediate impact of sensory removal while minimizing variability unrelated to the occlusion itself.

Gaze-based measures were sensitive to motion structure. A repeated-measures ANOVA on RMSE(Δ*v*_*G*_) across the three time-varying profiles (Linear, Sinusoidal, Semicircular; E_1_ cohort) revealed an effect of profile (*F*_(2,78)_ = 4.30, *p* = 0.017, partial *η^2^* = 0.099). Bonferroni-corrected pairwise contrasts showed that Linear differed reliably from Semicircular (*t*_(39)_ = 3.70, *p*_*Bonf*_ = 0.002, *dz* = 0.59), while the Linear–Sinusoidal and Sinusoidal–Semicircular contrasts did not survive correction (*p*_*Bonf*_ > 0.23). RMSE(GTD) showed much stronger profile dependence (*F*_(2,78)_ = 40.59, *p* < 0.001, partial *η^2^* = 0.510), confirming that spatial gaze error during occlusion was shaped substantially by the temporal structure of *v*_*T*_.

In contrast, manual speed control showed limited dependence on motion structure. RMSE(Δ*v*_*U*_) was lowest for constant speed and slightly higher for all time-varying profiles, but these differences did not reach statistical significance (all *p* > 0.09), and time-resolved analyses revealed no frame-specific effects surviving FDR correction. This indicates that manual speed matching during occlusion was relatively insensitive to the temporal structure of *v*_*T*_.

A repeated-measures ANOVA on RMSE(UTD) across the three time-varying profiles revealed a robust effect of profile (*F*_(2,78)_ = 70.4, *p* < 0.001, partial *η^2^* = 0.64). Planned pairwise contrasts (Bonferroni-corrected within metric) followed. Interception accuracy exhibited the strongest and most consistent effects. RMSE (UTD) was markedly lower for constant speed than for all other profiles (constant vs linear: *t*_(22)_ = −2.83, *p* = 0.010; constant vs sinusoidal: *t*_(22)_ = −2.55, *p* = 0.018; constant vs semicircular: *t*_(22)_ = −3.11, *p* = 0.005), with no differences among the non-constant conditions (all *p* > 0.58). Time-resolved analyses further revealed sustained model-dependent effects (28 of 80 frames surviving FDR correction), indicating progressive divergence in interception accuracy following masking. Average RMSE values for every metric and profile, together with the omnibus ANOVAs, are provided in Supplementary Figure 10.

To capture and compare these effects at a more “global” scale, we created an “overall performance metric” that integrates all dependent variables. For each measure, motion profiles were ranked according to the area under the curve (AUC) of the RMSE (lowest to highest), and ranks were averaged across variables to obtain a composite score. This score was then standardized to a higher-value index, with higher values indicating better overall performance (Figure 5B). To assess how these differences evolve over time, the metric was computed across progressively increasing frame windows within the occlusion interval.

Relative to constant speed, linear dynamics produced modest increases in manual speed error (RMSE(Δv_*U*_): +10.8%) but improved gaze-based measures, reducing both RMSE(Δv_*G*_) (−6.5%) and RMSE(GTD) (−13.4%). In contrast, sinusoidal and semicircular v_*T*_ profiles led to increases in cumulative error across most metrics, with particularly large degradations in interception accuracy (RMSE(UTD): ∼43–49%). Across dependent variables, linear speed profiles generally produced lower error values than constant-speed conditions, whereas nonlinear profiles did not yield comparable reductions in gaze or manual error. Counterintuitively, RMSE(Δv_*G*_) under masking was lower for linear ramps than for constant speed. A predictable, sign-flipping acceleration therefore gives gaze tracking a small but measurable advantage when visual input is removed, consistent with the matched-velocity result that acceleration information is present in the behavioral signal.

These findings indicated that predictive visuomotor control was not uniformly affected by occlusion but depended critically on the temporal structure of *v*_*T*_. Linear changes preserved local predictability and supported more accurate gaze-based tracking, whereas nonlinear dynamics imposed constraints that led to faster accumulation of error across both oculomotor and manual measures. Throughout these comparisons, observed interception rates remained well above a yoked-control benchmark (Supplementary Figure 3; see section “Materials and methods”), confirming that the effects of motion structure are differences in how participants intercept, not in whether they intercept above chance.

### Effects of acceleration- and deceleration-phase target masking on visuomotor interception

To further characterize the role of visual input during temporally structured motion, we used phase-locked masking, in which the target was intermittently removed during predefined temporal segments of the speed modulation cycle. Masking windows were positioned either on the acceleration phase of the cycle (E_2_; d*v*_*T*_/d*t* > 0) or on the deceleration phase (E_3_; d*v*_*T*_/d*t* < 0) and analyzed jointly. Unlike full masking, which removes visual input continuously, this manipulation allowed assessment of how brief, phase-locked interruptions affect tracking and interception, providing a more fine-grained test of how predictive mechanisms operate within ongoing *v*_*T*_ dynamics.

For linear speed ramps, phase-locked masking increased Δ*v*_*G*_ (*t*_(798)_ = −3.81, *p* < 0.001), and GTD (*t*_(798)_ = −3.48, *p* < 0.001), while Δ*v*_*U*_ did not differ from unmasked conditions (not illustrated). Despite stable speed matching, UTD increased with masking (*t*_(798)_ = 5.19, *p* < 0.001, *d* = 0.37), indicating reduced interception accuracy. Similar effects were observed for sinusoidal speed profiles: Δ*v*_*G*_ (*t*_(798)_ = −3.81, *p* < 0.001) and GTD (*t*_(798)_ = −3.48, *p* < 0.001) increased under masking, Δ*v*_*U*_ remained largely unchanged, and UTD showed a robust increase (*t*_(798)_ = 4.89, *p* < 0.001, *d* = 0.35; not illustrated).

Semicircular speed profiles produced the strongest effects. Phase-locked masking led to marked increases in Δ*v*_*G*_ and GTD (both *p* < 0.001), accompanied by substantial increases in UTD (*t*_(798)_ = 4.89, *p* < 0.001, *d* = 0.35), while Δ*v*_*U*_ again remained relatively stable (not illustrated). This indicates that transient visual interruptions are particularly disruptive when *v*_*T*_ varies nonlinearly over time.

Thus, across v_*T*_ profiles, phase-locked masking primarily disrupted gaze tracking and interception accuracy, with increases in Δv_*G*_ and GTD and a robust deterioration in UTD, while Δv_*U*_ remained comparatively stable. Notably, UTD showed the strongest dependence on both v_*T*_ profile and temporal frequency, indicating that interception accuracy was the most sensitive measure of disruption under intermittent occlusion. These results demonstrate that brief, phase-locked losses of visual input are sufficient to impair performance, particularly when v_*T*_ varies nonlinearly, revealing that predictive control remains continuously engaged yet vulnerable to transient disruptions within the evolving speed signal.

## Saccade-exclusion sensitivity

At 60 Hz sampling rate, instantaneous gaze velocity is sensitive to saccade frequency and amplitude, so a masking-induced change in Δ*v*_*G*_ could, in principle, reflect altered saccade kinematics rather than altered gaze tracking. To rule this out, we re-ran the main analyses after excluding frames with instantaneous |*v*_*G*_| > 100°/s (plus ± 1 frame of padding). Saccade-frame fractions averaged 13–14% across profiles and differed across speed profiles and visibility (Profile × Visibility: *F*_(2,78)_ = 17.76, *p* < 0.001, partial *η^2^* = 0.31). After exclusion, every masked-vs-unmasked contrast remained significant in the same direction, with larger effect sizes than the original (Δ*v*_*G*_: *dz* = 1.7–2.1 after exclusion vs 0.7–1.2 in the original; GTD: *dz* > 3.0 across profiles). At the participant level, GTD was essentially identical with or without saccade exclusion (Pearson *r* > 0.99 in every condition), confirming that the spatial result does not depend on event classification. The Δ*v*_*G*_ magnitudes shifted (Pearson *r* ≈ 0.45–0.54 between versions), consistent with saccades contributing noise to instantaneous gaze velocity but not to the direction of the effect. Full side-by-side statistics appear in Supplementary Figure 2 and Supplementary Table 3.

### Pupillary dynamics during visuomotor interception

**FIGURE 6:**
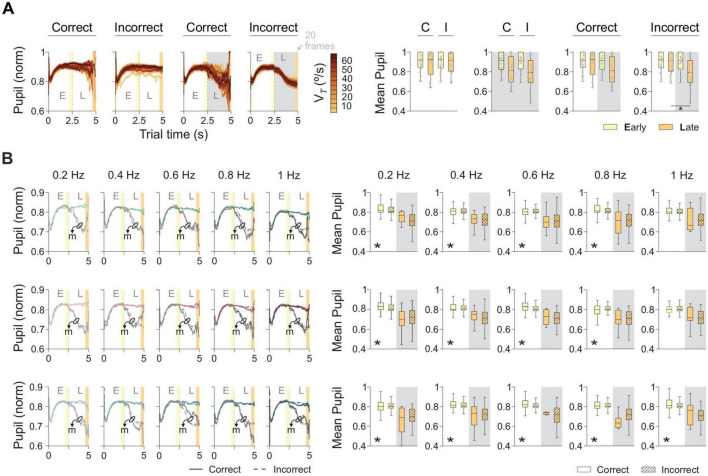
Pupillary dynamics during interception. **(A)** Group-average pupil size over time for the constant *v*_*T*_ condition, separated by correct and incorrect trials under unmasked (left) and masked (right) conditions. Gray shaded regions indicate the masking period, while colored shaded regions denote the early (E) and late (L) analysis windows. Adjacent whisker plots summarize pupil size within these epochs as a function of trial outcome and visibility condition. **(B)** Group-average pupil traces for time-varying *v*_*T*_ profiles: linear ramps (top), sinusoidal (middle), and semicircular (bottom). Traces from masked and unmasked conditions are overlaid, with correct trials shown as solid lines and incorrect trials as dotted lines. Traces corresponding to the masked condition are additionally highlighted by a black ring and an “m” for visual identification. Whereas pupil size remained relatively stable in unmasked condition throughout the trial, masked trials exhibited a pronounced reduction in pupil size following mask onset (vertical dotted line). Whisker plots summarize pupil size during the late analysis window across temporal frequencies, separated by correctness and visibility condition. Across profiles, pupillary modulation emerged primarily after mask onset and showed little dependence on the specific form of target speed dynamics. Asterisks in the whisker plots denote significantly lower late pupil diameter in the masked relative to the unmasked condition.

We analyzed pupillary responses as an exploratory complement to the gaze and manual metrics. Average pupil size per participant was extracted in two windows, an early window (“E” frames 131–150, before masking onset) and a late window (“L” frames 281–300, near trial end), and analyzed per profile with a 2 × 2 repeated-measures ANOVA (window × visibility). Group-average pupil traces for the constant-*v*_*T*_ condition are shown in Figure 6A; group-average pupil traces for the three time-varying profiles (linear ramps, sinusoidal, semicircular) are shown in Figure 6B; per-profile interaction plots and the outcome and gaze-locus splits are provided in Supplementary Figure 11. Across all four speed profiles, the window × visibility interaction was robust (Constant: *F*_(1,39)_ = 115.1, *p* < 0.001, partial *η^2^* = 0.75; Linear: *F*_(1,39)_ = 163.3, *p* < 0.001, partial *η^2^* = 0.81; Sinusoidal: *F*_(1,39)_ = 179.0, *p* < 0.001, partial *η^2^* = 0.82; Semicircular: *F*_(1,39)_ = 172.5, *p* < 0.001, partial *η^2^* = 0.82). Bonferroni-corrected planned contrasts located the effect in the late window: in every profile, late-masked pupil was lower than late-unmasked (all |*t*_(39)_| > 10.9, *p*_*Bonf*_ < 10^–2^), whereas early-masked and early unmasked did not differ (all *p*_*Bonf*_ ≥ 0.54). The pattern is consistent across motion regimes and did not depend on whether the speed profile was constant, linear, or nonlinear.

Splitting masked trials by outcome (see section “Materials and methods”), the window × visibility interaction held for both correct and incorrect trials (correct: *F*_(1,11–30)_ between 8.4 and 28.7, all *p* < 0.013; incorrect: *F*_(1,39)_ between 136.2 and 186.3, all *p* < 10^−13^). The effect was larger in incorrect trials, suggesting that pupil reduction tracks the engagement of internal estimates more strongly when those estimates fail to support successful interception.

A potential confound could be the small change in mean screen luminance when the target disappears. Although geometric considerations make this contribution small (the target subtends ∼4.5 × 10^−5^ of the visible screen area; estimated mean-luminance change ≈ 0.002 cd/m^2^ against a 50 cd/m^2^ background), we tested the luminance contribution empirically. We split late-window frames by whether gaze was within 4° of the (still-tracked) target position. The relevant test is the visibility × gazelocus interaction: under a local-luminance account, the pupil reduction under masking should be larger when gaze was near the disappearing target. The interaction was not reliable in any profile (Constant: *F*_(1,39)_ = 0.02, *p* = 0.90; Linear: *F*_(1,39)_ = 0.01, *p* = 0.94; Sinusoidal: *F*_(1,39)_ = 0.44, *p* = 0.51; Semicircular: *F*_(1,39)_ = 1.04, *p* = 0.31). The Visibility main effect remained strong across all locus conditions (all *F*_(1,39)_ > 108, all *p* < 10^–2^). The pupil reduction under masking therefore did not depend on where gaze fell when the target disappeared. This rules out a local-luminance account and points instead to a cognitive one: the late-window pupil tracked the gain assigned to internal estimates once sensory input was removed (Mathôt and Vilotijević, 2023; Vincent et al., 2019).

## Discussion

We examined how visuomotor interception adapts to changes in the temporal structure of *v*_*T*_ and how those adaptations depend on the availability of visual feedback. Across all conditions, behavior remained relatively coupled to target motion, even when visual input was transiently removed. However, this coupling was not uniformly robust: its stability depended strongly on how *v*_*T*_ evolved over time, revealing clear constraints on predictive control.

Under constant velocity, interception was comparatively stable. Both gaze and manual responses scaled with *v*_*T*_, yet showed speed-dependent biases: overshooting at low velocities and undershooting at higher ones, with distinct transition points across effectors. These differences are consistent with the view that oculomotor and manual control regimes operate partially independently rather than reflecting a single shared estimate (Orban de Xivry et al., 2013; Spering and Montagnini, 2011). Masking did not abolish the relationship between behavior and target motion. Although spatial errors increased and speed matching degraded, the underlying structure of responses persisted. Velocity representations were maintained over short intervals and used to guide behavior in the absence of continuous input (Brenner and Smeets, 2018; Wolpert et al., 1995).

This pattern is consistent with the broader literature, which shows that interception relies predominantly on velocity information. More specifically, observers tend to behave as if motion continues at its last observed speed, even when acceleration is present (Benguigui et al., 2003; Brouwer et al., 2002; Tresilian, 1999). Predicting position from velocity is computationally cheap relative to estimating higher-order dynamics from sparse sensory samples, so this could reflect a strategy, not a failure of cognition. Our data, however, refine this picture in two converging ways. First, the within-cycle matched-velocity test was unambiguous: at the same instantaneous *v*_*T*_, behavior differed reliably between acceleration and deceleration across every metric and every velocity bin (10/10 bins FDR-significant in each of Δ*v*_*G*_, GTD, Δ*v*_*U*_, and UTD). Second, when speed varied as a periodic linear ramp, gaze-based RMSE under masking was lower than under a constant *v*_*T*_. Both observations point in the same direction: acceleration-related information is present in the behavioral signal, and the system uses it, even if velocity remains the dominant variable in coarser averages.

When speed varied linearly, the cost fell selectively on spatial accuracy. Speed matching stayed stable across ramp frequencies, which points to continuous local updating rather than an explicit acceleration term entering the speed-matching signal itself. The within-cycle contrast described above tells a complementary story: at matched instantaneous *v*_*T*_, behavior still diverged between acceleration and deceleration. Acceleration-related information is therefore present in the predictive computation, even if it does not enter speed matching strongly enough to neutralize the cost of nonlinear speed structure. This fits neurophysiological evidence that motion-sensitive cortical areas carry acceleration-related signals during smooth pursuit (Lisberger and Movshon, 1999), manual reaching studies in which acceleration- and deceleration-phase responses diverge (de Brouwer et al., 2002), and model-based work showing that the brain uses the recent history of target motion rather than the instantaneous sample alone (Bosco et al., 2015).

We do not interpret this as evidence against velocity-based extrapolation under occlusion: the local approximation likely dominated during brief masking on locally linear segments. However, under our design, where motion was varied across structures and observers tracked for several seconds, first-order signals alone were insufficient. Once *v*_*T*_ varied nonlinearly, this partial exploitation of acceleration was no longer enough. Sinusoidal and semicircular profiles imposed time-varying acceleration that cannot be captured by either a constant-velocity or a constant-acceleration approximation, and performance degraded more strongly under masking, exactly the pattern expected if the system carries acceleration-related information but only the sign, not the time course, of acceleration. Behavior nonetheless remained reliably above a yoked-control random-policy benchmark across all conditions. The benchmark estimated what an unrelated cursor trajectory would have scored against the same targets, and real performance exceeded that null in every participant and condition. Even so, both spatial error and gaze–target misalignment increased with modulation frequency. Previous work has shown that visuomotor behavior can entrain to smoothly varying speed signals when continuous feedback is present (Treviño and Márquez, 2025a). The results presented here show that this entrainment broke down once visual input was interrupted: prediction alone did not sustain accurate tracking. A parsimonious interpretation is that predictive mechanisms relied on locally valid approximations of target velocity, with the behavioral signal also carrying the sign of acceleration; errors accumulated rapidly once speed changes could no longer be approximated as locally linear (Brenner et al., 2016; Kreyenmeier et al., 2021).

Placed in a broader context, these findings converge with a consistent pattern across interception paradigms. When motion is occluded, both eye and hand often rely heavily on the most recently available velocity sample. In eye–hand coordination tasks with accelerating targets, interception timing is best explained by a “final velocity” strategy rather than by models incorporating higher-order motion signals (Kreyenmeier et al., 2022). Our data fit this velocity-dominated picture but qualify it: at matched instantaneous speed, the sign of acceleration still biased behavior, so first-order extrapolation captures the dominant trend without being the whole story. When internal priors are violated, as in perturbed gravitational motion, predictions become unstable, leading to large spatial and temporal errors during occlusion (Bosco et al., 2012).

More generally, as motion departs from simple, predictable dynamics, behavior probably shifts toward greater reliance on continuous sensory input. Increased variability, nonlinear forces, or masking all degrade predictive performance and promote feedback-driven control (Márquez et al., 2024; Zhao and Warren, 2013). Even predictive gaze strategies, which typically anticipate future target states, are disrupted when visual information is limited or when the target’s speed changes unfold over very short time windows (Treviño and Márquez, 2025b). Across paradigms, a similar constraint emerges: prediction is very effective when motion can be approximated locally by velocity but degrades sharply when this approximation breaks down.

The dissociation between gaze and manual behavior points to where a limit on predictive control sits. Three observations converge on this. First, under constant *v*_*T*_, the over- and undershoot transitions in gaze occurred at lower target speeds than in manual control, indicating that the two effectors did not share a single internal speed estimate. Second, removing visual feedback increased per-speed gaze-based errors at almost every constant speed tested, while manual speed error reached significance only at the highest speeds. Third, under masking with nonlinear speed profiles (sinusoidal and semicircular), gaze-based RMSE depended on motion profile, while manual speed RMSE did not. Together, these observations indicate that oculomotor behavior operated closer to the sensory sampling process, continuously updating motion estimates, while manual control probably relied on more stable, temporally integrated signals (Cámara et al., 2018; Kreyenmeier et al., 2021). Rather than reflecting a single predictive model, visuomotor control appears distributed across subsystems with distinct temporal and functional constraints (Márquez and Treviño, 2026).

Visual feedback stabilized performance across conditions. Its removal increased spatial errors across all conditions, but the magnitude of this effect depended on motion structure. Constant and linear *v*_*T*_ trajectories showed relatively modest degradation, whereas nonlinear *v*_*T*_ dynamics produced greater impairments. This dependence reinforces the idea that prediction is not uniformly flexible but constrained by the representational format of motion, with velocity occupying a privileged role (Brouwer et al., 2002; Kreyenmeier et al., 2021; Tresilian, 1999). One reading, consistent with our data though not established by them, is that internal forward models keep behavior roughly on track while sensory input is briefly gone, with error building up because no online correction is possible during occlusion (Franklin and Franklin, 2023; McKenna et al., 2017).

Pupillometry provided convergent evidence for the behavioral findings. Across all speed profiles, pupil size decreased following target masking, whereas no differences were observed before the target disappeared. This effect was present in both successful and unsuccessful trials but was more pronounced when participants failed to intercept the target, suggesting that pupil dynamics tracked the quality of the internal estimate supporting behavior. Importantly, the reduction is unlikely to be explained by changes in luminance. The target occupied a negligible portion of the display, and additional analyses showed that the effect did not depend on gaze position relative to the disappearing target. Pupil size decreased regardless of whether participants were looking near or far from the target at the time of occlusion. Therefore, this response pattern is inconsistent with a local pupillary light reflex and instead suggests that the observed modulation reflects changes in cognitive processing associated with prediction and target tracking after visual information becomes unavailable.

The late-window reduction is consistent with the predictive-processing account that pupil size tracks the precision assigned to internal estimates when sensory input is unreliable (Friston, 2010; Márquez and Treviño, 2024a; Mathôt and Vilotijević, 2023; Vincent et al., 2019). The outcome-dependent modulation, evident even across trials with the same visual conditions during masking, is the clearest cognitive signature in the pupil data. Pupillary responses showed little dependence on *v*_*T*_’s temporal structure, remaining comparable across linear and nonlinear profiles. We interpret this as evidence that pupil dynamics reflect global task demands and the internal-estimate gain, rather than the specific motion parameters that drove the gaze and manual metrics (Márquez and Treviño, 2024a).

At the neural level, the combined picture (velocity-based prediction that survives masking, plus a smaller acceleration-related signal that biases behavior at matched velocity) is consistent with known properties of motion processing. Cortical areas such as MT and MST encode velocity robustly and can support predictive tracking over short occlusions, whereas representations of acceleration are weaker and could emerge indirectly through temporal integration (Tsutsui et al., 2021). That difference offers a mechanistic explanation for why velocity-based prediction remains stable under masking while higher-order dynamics require continuous input. More broadly, these findings align with frameworks in which internal models prioritize dominant environmental statistics over encoding the full generative structure (Shadmehr et al., 2010; Wolpert and Flanagan, 2016).

The dependence of performance on *v*_*T*_ further highlights a fundamental constraint. When the interceptor has a large speed advantage, reactive strategies are sufficient, allowing continuous correction toward the target. As this margin decreases, sensorimotor delays become limiting, and behavior shifts toward anticipatory control (Brenner and Smeets, 2018; Márquez and Treviño, 2026). Under these conditions, the effectiveness of prediction depends critically on whether motion can be approximated using low-order representations. The present results suggest that this approximation defines the operational boundary of predictive interception.

These constraints have direct implications beyond laboratory settings. Aging, for instance, is associated with increased sensory noise and a stronger reliance on predictive signals. Older adults show enhanced motion-induced position shifts, consistent with increased weighting of motion-based extrapolation when spatial estimates are unreliable (Jeon et al., 2020). In interception contexts, this is accompanied by earlier and more sustained predictive gaze strategies, likely compensating for slower processing but potentially increasing vulnerability to model violations (Gerharz and Voudouris, 2025). Similarly, neurological conditions that disrupt timing, sensory integration, or internal model calibration, such as Parkinson’s disease or multiple sclerosis, are likely to further impair predictive control, particularly under occlusion or nonlinear dynamics (Bonanno et al., 2025; Lefebvre et al., 2025). In contrast, conditions such as Alzheimer’s disease may reduce the contribution of predictive motion signals altogether, reflecting a breakdown in the underlying representations (Jeon et al., 2020). Together, these observations suggest that the balance between prediction and feedback is not fixed but could shift systematically with changes in sensory reliability and neural function.

Several limitations of this work should be acknowledged. The controlled trajectories used here, like natural object motion, share the property that *v*_*T*_ varies systematically over time, which is the dimension relevant to our predictive-control question. However, they differ from natural motion in three respects. First, they lack 3D depth cues and aerodynamic curvature. Second, they do not engage the vestibular–cortical prior that is known to bias predictions for free-falling objects (Delle Monache et al., 2023; Zago et al., 2004). Third, the 2D arena removes the binocular disparity signal that supports time-to-contact estimation in catching tasks. The findings here, therefore, apply to motion regimes in which prediction must operate without strong priors about the generative source, and we do not extend them to free-fall, depth-rich, or binocular-disparity-rich conditions. Extending the paradigm to 3D dynamics with parabolic or vortical trajectories would test the same local-velocity hypothesis under stronger competing priors. Electrophysiological recordings will also be required to determine how predictive representations are instantiated and coordinated across oculomotor and motor control.

## Conclusion

Predictive visuomotor interception remained functional in the absence of continuous visual input, but its effectiveness was constrained by the temporal structure of *v*_*T*_. The visuomotor system relied predominantly on velocity-based representations that supported stable performance when speed was constant or changed linearly over time; under nonlinear speed variations, the same representations were insufficient, masking under sinusoidal and semicircular profiles produced markedly larger gaze and interception errors than under constant or linear *v*_*T*_ ramps, indicating that the locally valid approximation no longer held. The matched-velocity test on the linear-ramp data, however, showed that it is not insensitive to acceleration: behavior at the same instantaneous speed differed systematically between acceleration and deceleration. The operational boundary of predictive interception, therefore, is not set by a strict velocity-only representation, but by the limited precision with which higher-order kinematic information is exploited, sufficient to bias behavior at matched velocity, but insufficient to prevent error accumulation under nonlinear masking.

Pupillary dynamics complement this picture. The absence of early differences and the emergence of modulation during masked intervals indicate that pupil size tracks the transition from externally guided behavior to reliance on internal estimates. The late-window reduction under masking, larger on incorrect trials, and independent of gaze position at the moment of target disappearance, is consistent with a “cognitive” interpretation in which pupil size indexes the precision assigned to internal estimates when sensory input is unreliable.

Together, these results indicate that predictive interception is not supported by a fully general motion representation (Márquez and Treviño, 2025), but rather by velocity-dominated representations whose reliability depends on the structure of the sensory input and the interceptor’s current state. A clear, testable next step is to ask whether these constraints ease when stronger generative priors, gravity, depth, and binocular disparity are available, that is, whether the velocity-dominated regime described here is a property of the visuomotor system itself or a property of motion regimes in which no stronger prior applies.

## Data Availability

The datasets presented in this study can be found in online repositories. The names of the repository/repositories and accession number(s) can be found below: The datasets used for this study can be found in the OSF repository: https://osf.io/qrh79/overview?view_only=1598ae2a560d459e979bc6b420883f18.
