# Supplementary material for: Predictive interception across speed profiles
Source: Front Neural Circuits. 2026 Jul 13;20:1848603. doi: 10.3389/fncir.2026.1848603 (PMC13402429; doi:10.3389/fncir.2026.1848603)
Supplement: Supplementary file 1 [file Data_Sheet_1.pdf]

Supplementary Material

Predictive Interception across Speed Profiles

Inmaculada Márquez[1,2,3], Mario Treviño[1*]

[1]Laboratorio de Plasticidad Cortical y Aprendizaje Perceptual, Instituto de Neurociencias, Universidad de Guadalajara, Guadalajara, Jalisco, México

[2]Departamento de Psicología, Centro Universitario de la Ciénega, Universidad de Guadalajara, Ocotlán, México

[3]Laboratorio de Neurofisiología, Departamento de Bioingeniería Traslacional, Centro Universitario de Ciencias Exactas e Ingenierías, Guadalajara, Jalisco, México

*** Correspondence:**

Dr. Mario Treviño (mario.trevino@academicos.udg.mx), Laboratorio de Plasticidad Cortical y Aprendizaje Perceptual, Instituto de Neurociencias, Universidad de Guadalajara. Francisco de Quevedo 180, Arcos Vallarta. C.P. 44130. Guadalajara, Jalisco, México.

Keywords: Target speed; target acceleration; sensory prediction errors; visuomotor control; eye tracking; visual masking

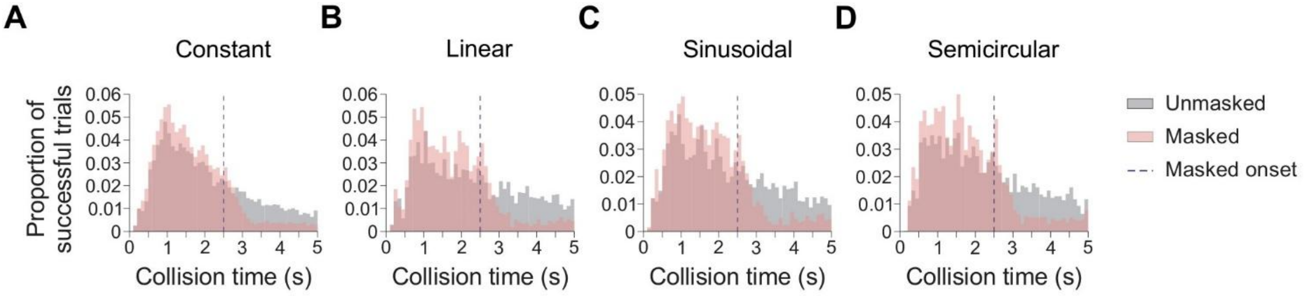

Supplementary Figure S1. Collision-time distributions per speed profile. Most successful interceptions complete before the masking onset. Histograms of collision time (CT) on successful trials, per profile (Constant (A), Linear (B), Sinusoidal (C), Semicircular (D)), with unmasked (gray) and masked (red) trials overlaid as proportions. Vertical dashed line: masking onset at 2.5 s. Bin width: 0.1 s. Panel titles report, per profile and visibility, the total number of trials, the success rate (percentage of trials ending in successful interception), the mean CT ± SD, and (for masked trials only) the fraction of successful trials terminating before the masking onset. Across the 60,000-trial dataset, the overall success rate was 66.0%. Mean CTs on successful unmasked trials were 2.04 s (Constant), 2.28 s (Linear), 2.22 s (Sinusoidal), and 2.20 s (Semicircular). Mean CTs on successful masked trials clustered at 1.7–1.8 s across profiles. Between 79% and 83% of successful masked trials were completed before the masking onset and are therefore unaffected by the visual manipulation; these trials are excluded from masked-window analyses (see **Methods** and **Supplementary Table S2**).

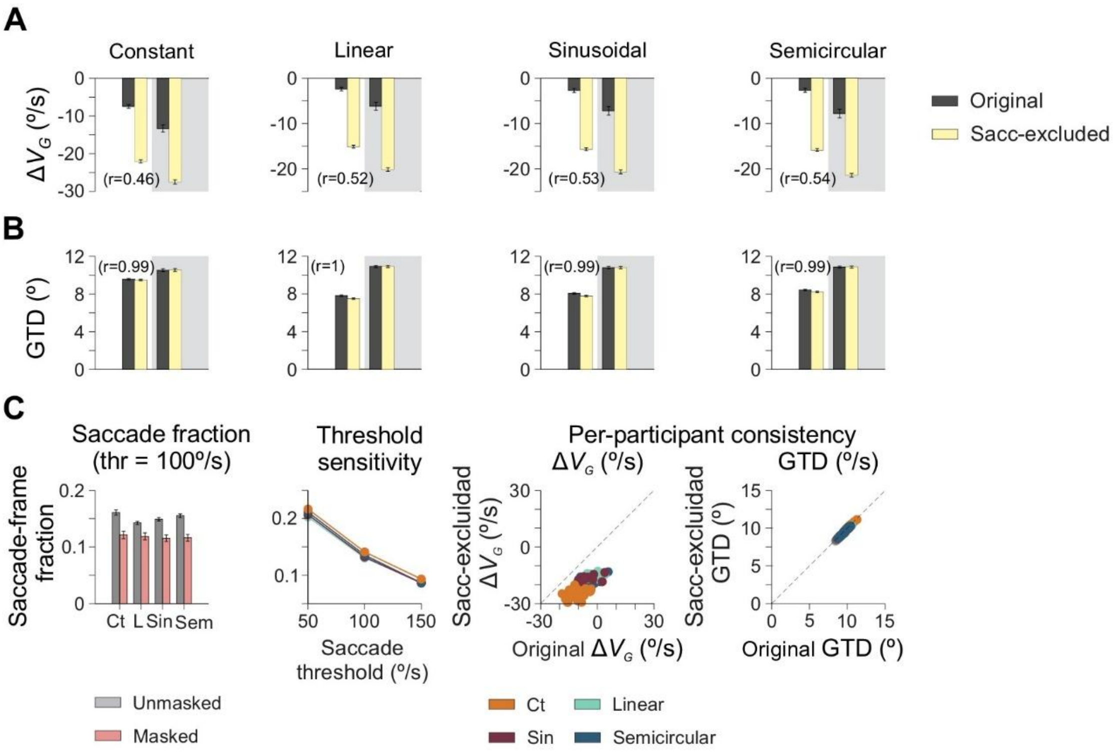

Supplementary Figure S2. Saccade-exclusion sensitivity preserves the masking effects on gaze velocity and gaze–target distance. Headline gaze-based analyses (Δv_G and GTD; masked vs unmasked) re-computed with and without saccade-like frames excluded. Saccade frames were defined as $|v_G| >$ 100°/s with ±1-frame padding; trials with more than 50% saccade-tagged frames in the 2.5–5 s analysis window were dropped from the saccade-excluded version. (A) Δv_G per profile (Constant, Linear, Sinusoidal, Semicircular), original (gray) versus saccade-excluded (orange) bars, masked and unmasked side by side. (B) Same for GTD. (C) Left: saccade-frame fraction per profile and visibility. Middle: threshold sensitivity (saccade-frame fraction at 50, 100, 150°/s thresholds, per profile). Right two panels: per-participant scatter of original vs saccade-excluded Δv_G and GTD, with the y = x reference line. Title strings on each top-row and middle-row panel report the Pearson *r* between original and saccade-excluded per-participant means, pooled across visibility. GTD is robust to saccade exclusion at the participant level (Pearson $r > 0.99$ across all four profiles); Δv_G correlations are lower ($r \approx 0.45$–0.54), consistent with saccades contributing instantaneous-velocity noise but not changing the direction of the masking effect. $n = 40$ participants per profile; 2-way RM-ANOVA on saccade-frame fraction across the three speed profiles × visibility revealed reliable main effects of speed profile ($F_{(2,78)} = 9.18$, $p < 0.001$, partial $\eta^2 = 0.19$) and visibility ($F_{(1,39)} = 32.35$, $p < 0.001$, partial $\eta^2 = 0.45$), and an interaction ($F_{(2,78)} = 17.76$, $p < 0.001$, partial $\eta^2 = 0.31$). Full side-by-side statistics in **Supplementary Table S2**.

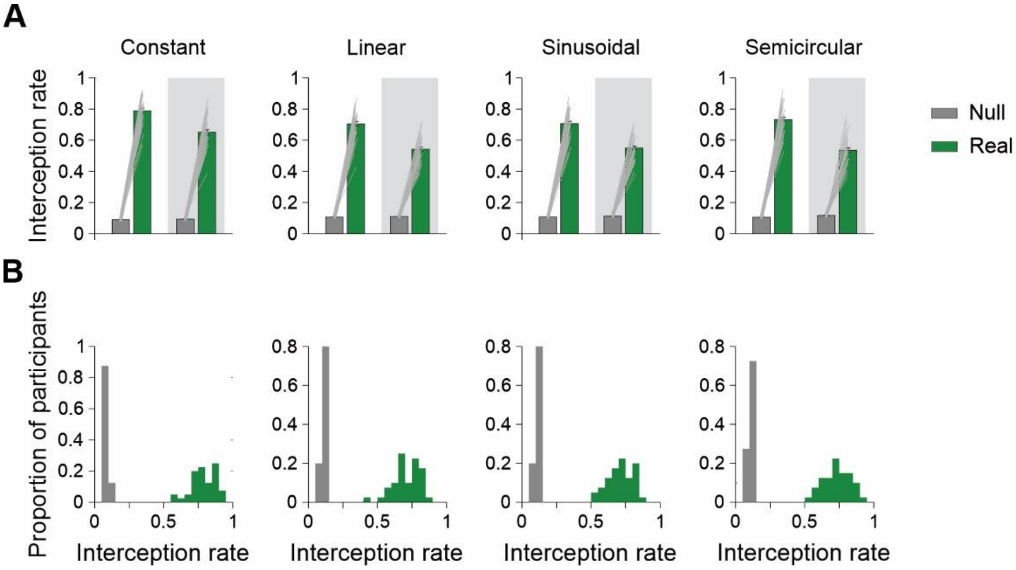

Supplementary Figure S3. Yoked-control random-policy benchmark. Real interception rates exceed a yoked-control random-policy benchmark in every condition. Per (profile × visibility) cell, comparison of real interception rates against a null distribution obtained by yoking each participant's cursor trajectories to target trajectories drawn from other trials within the same cell and replaying the scoring procedure. Scoring threshold: 0.59° (empirical 95th percentile of cursor–target distance at successful interception). 500 yoking permutations per cell. (A) Per-profile bar plots showing mean interception rate (null, gray; real, green) for unmasked and masked conditions side by side, with thin lines connecting each participant's null and real rates. Error bars: SEM across n = 40 participants. Panel-level annotations: paired t-test statistic and Cohen's dz above each pair. Bottom row: per-profile distributions across participants, normalized histograms of real (green) and null (gray) interception rates for the unmasked condition. All 320 participant × cell combinations exceeded the 95th percentile of their own null distribution (100% in every cell). Real-vs-null differences ranged from 0.42 to 0.70 across cells (paired $t_{(39)}$ = 27.22–44.98; p < 10−26 in every cell). "Above-chance" performance in the main text refers to this benchmark.

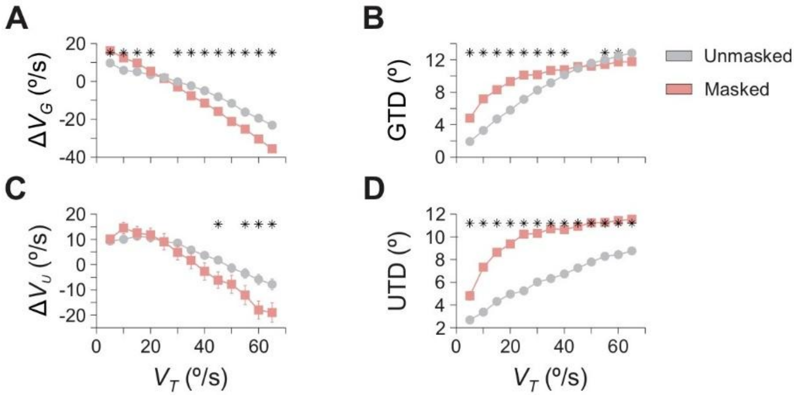

Supplementary Figure S4. Speed-resolved masking effects under constant target velocity. Gaze-based and spatial measures are broadly affected; manual speed is selectively affected at higher target velocities. Per-speed contrasts (masked *vs* unmasked, paired *t*-test) at each of the 13 constant target velocities (5–65°/s in 5°/s steps), for the four behavioral metrics: Δv_G (A), GTD (B), Δv_U (C), UTD (D). One panel per metric. Within each panel, unmasked (gray circles) and masked (red squares) per-participant means ± SEM plotted against v_T. Asterisks above markers: bins with FDR-corrected $p < 0.05$ (Benjamini–Hochberg across the 13 tests per metric). Panel titles report the number of FDR-significant speeds per metric: 12/13 for Δv_G (non-significant: 25°/s), 11/13 for GTD (non-significant: 45°/s, 50°/s), 4/13 for Δv_U (significant only at 45, 55, 60, 65°/s), and 13/13 for UTD. The dissociation between Δv_U (low FDR-significant count) and the gaze-based plus spatial metrics (high FDR-significant count) parallels the eye/hand asymmetry reported in the time-varying conditions. Constant-v_T cohort (E_4), $n = 40$ participants. Full per-speed statistics in **Supplementary Table S4**.

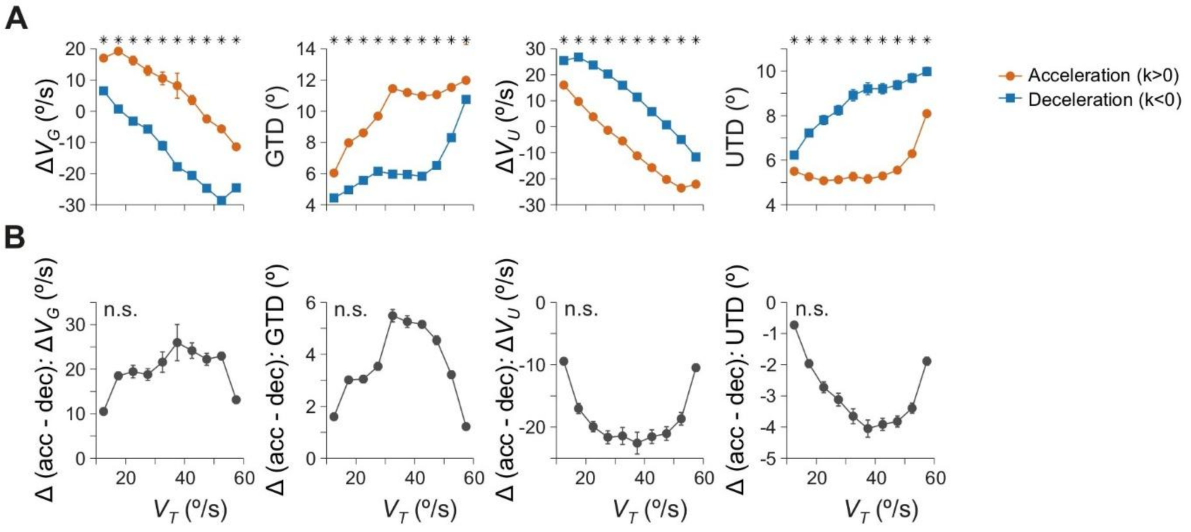

Supplementary Figure S5. Acceleration and deceleration produce different behavior at matched instantaneous target velocity. For each frame of the linear-ramp condition, the instantaneous target velocity v_T was binned into 5°/s intervals (10 to 60°/s; 10 bins). Within each bin, behavior was separated by cycle phase: acceleration ($dv_T/dt > 0$; orange) and deceleration ($dv_T/dt < 0$; teal). (A) Per-bin means of Δv_G (gaze speed minus target speed) and GTD (gaze–target distance) plotted against v_T. Bottom row: same for Δv_U (manual speed error) and UTD (cursor–target distance). (B) Per-participant paired differences (acceleration minus deceleration) per bin, with the Bayes factor BF01 printed above each bin (JZS Cauchy prior, $r = \sqrt{2}/2$). Asterisks mark bins with FDR-corrected $p < 0.05$ (Benjamini–Hochberg across the 10 bins per metric). Error bars: SEM across $n = 40$ participants (cohort E_1). Unmasked trials only. Eye and Hand tags identify gaze-derived and manual-derived metrics. All 40 bins (4 metrics × 10 bins) survived FDR correction; BF01 < 1 across all bins indicates the data favor a phase effect over the null. Per-bin statistics in **Supplementary Table S5**.

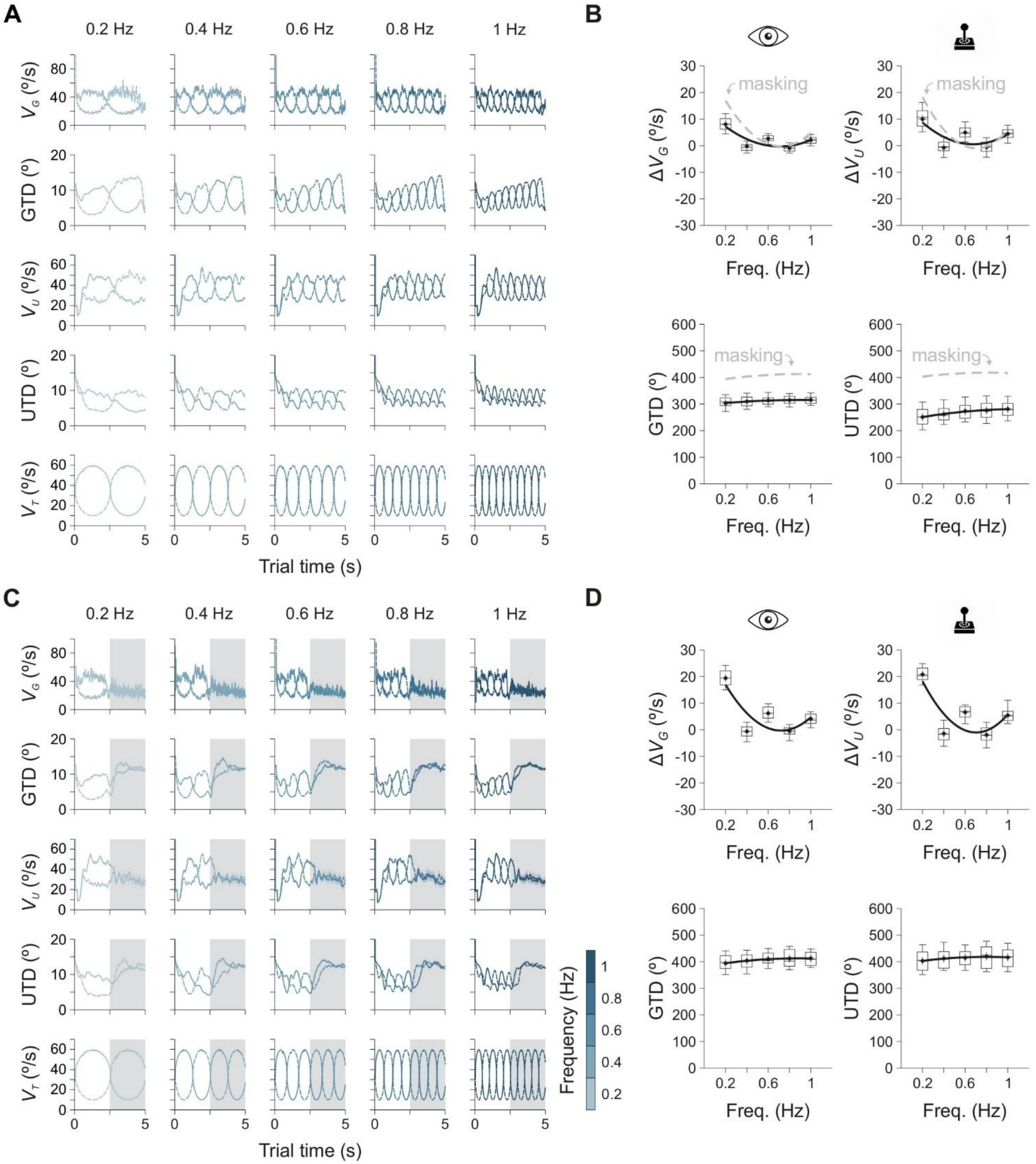

Supplementary Figure S6. Interception under Semicircular Speed Modulation. (A) Group-average traces of gaze and manual behavior during periodic semicircular changes in target speed (v_T) across temporal frequencies (left to right). Bottom row shows the imposed semicircular v_T profiles. (B) Summary metrics computed during the analysis window (2.5–5 s): Δv_G, GTD, Δv_U, and UTD as functions of modulation frequency. Lines indicate best-fitting models (linear or quadratic). Overlaid

gray dotted lines represent fits obtained under masking conditions for comparison. (C) Same analyses under masking (gray patch indicates the occlusion window). (D) Summary metrics under masking illustrating changes induced by loss of visual input.

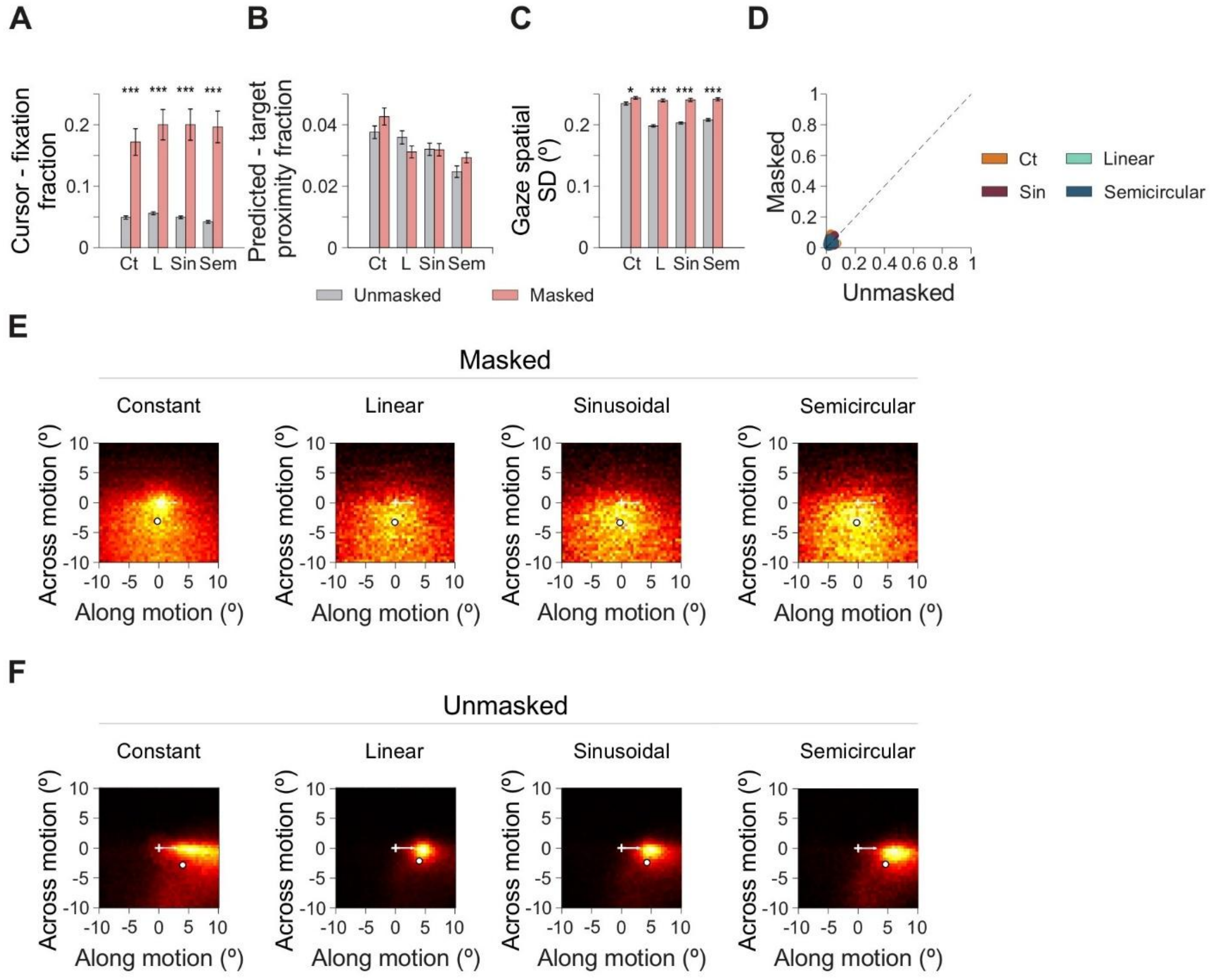

Supplementary Figure S7. Behavioral strategy during masking. Gaze re-organizes around the predicted target trajectory rather than monitoring the cursor. Per-trial measures computed across the 100 frames following masking onset, separated by speed profile (Constant, Linear, Sinusoidal, Semicircular) and visibility (unmasked, masked). (A–C) Bar plots of cursor-fixation fraction (gaze within 2° of cursor), predicted-target proximity fraction (gaze within 2° of the still-tracked target), and gaze spatial standard deviation around the expected target path. Bars: per-participant means ± SEM (n = 40). Asterisks above bars: Bonferroni-corrected paired t-test (masked vs unmasked), * $p < 0.05$, ** $p < 0.01$, *** $p < 0.001$. (D) Per-participant scatter of predicted-target proximity fraction (unmasked vs masked), color-coded by profile; dashed line is $y = x$. (E) 2D gaze density heatmaps for the masked condition, one panel per profile. Coordinate frame centered on the expected target position; x-axis aligned with the instantaneous target velocity direction ("along motion"). White cross: expected target location. White circle: centroid of gaze samples. White arrow: motion direction. Color: normalized sample density. (F) Same maps for unmasked trials. Cursor-fixation fraction increased under masking (4.2–5.6% unmasked *vs* 17.2–20.0% masked; dz = 0.89–0.95 across profiles; all $p_{Bonf} < 10-5$) but remained well below the predominant-strategy threshold. Gaze spatial SD increased under masking, more strongly for time-varying profiles (Linear: 7.9 → 9.6°, dz = 2.36; Sinusoidal: 8.1 → 9.6°, dz = 1.88; Semicircular: 8.3 → 9.7°, dz = 1.80; all $p_{Bonf} < 10-12$) than for Constant (9.4 → 9.8°, dz = 0.44, $p_{Bonf} = 0.033$). Profile × Visibility interaction on gaze SD: $F_{(2,78)} = 3.31$, $p = 0.042$, partial $\eta^2 = 0.078$.

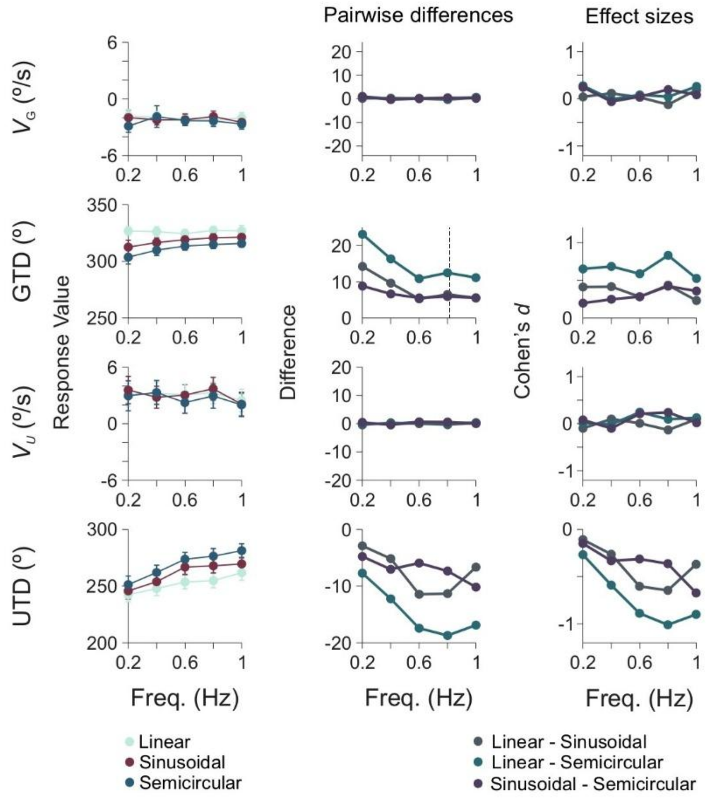

Supplementary Figure S8. Performance comparison across speed profiles. Within-subject comparison across linear, sinusoidal and semicircular speed profiles. Left column: group-average behavioral metrics (Δv_G, GTD, Δv_U and UTD) as functions of temporal frequency (2.5–5 s window). Linear v_T in teal, sinusoidal in pink, semicircular in blue. Middle column: pairwise differences between profiles ([Linear–Sinusoidal] in gray; [Linear–Semicircular] in dark teal; [Sinusoidal–Semicircular] in purple). Right column: corresponding effect sizes (Cohen's dz) using the same colour code. All conditions share matched speed ranges. Differences reveal frequency-dependent divergence in behavioral performance across motion structures.

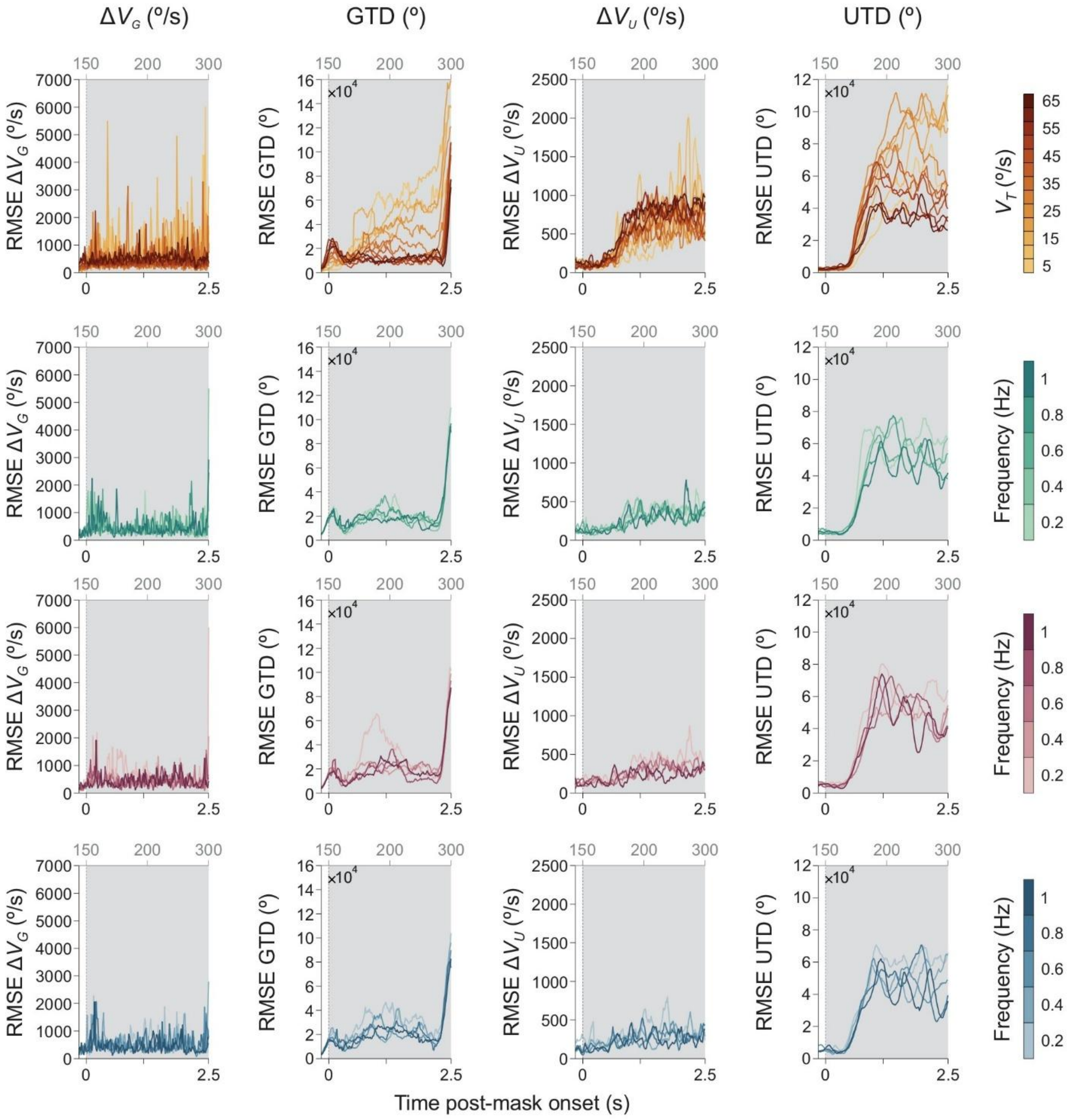

Supplementary Figure S9. Time-resolved masking-induced errors. Group-average RMSE time courses for Δv_G, GTD, Δv_U and UTD during the masking window (2.5–5 s). Columns: behavioral metrics; rows: speed profiles (constant, linear, sinusoidal, semicircular). Error magnitude increases over time and differs across motion structures, with nonlinear profiles exhibiting earlier and steeper error growth. This figure provides the temporal basis for the analyses summarised in **Figure 5**.

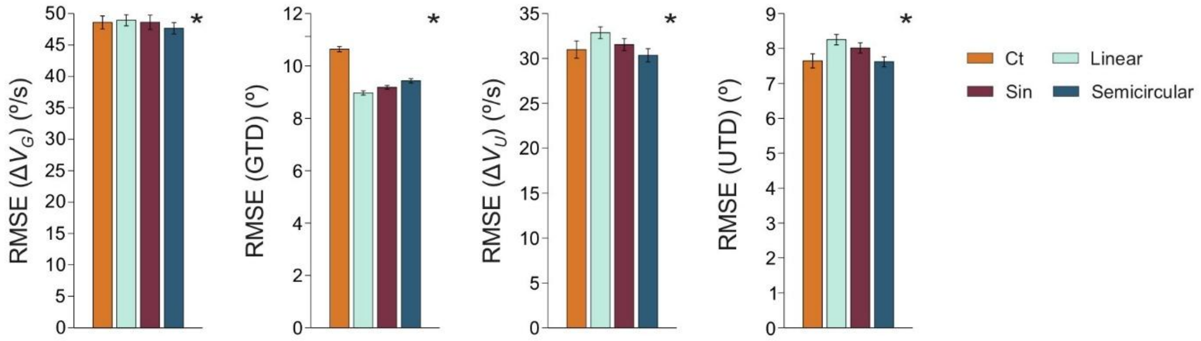

Supplementary Figure S10. Per-participant RMSE per metric per profile, computed on unmasked trials only. Within each trial and the 2.5–5 s analysis window, RMSE was computed as $\sqrt{}$mean(metric²): for Δv_G and Δv_U the metric is the signed difference from vT; for GTD and UTD the metric is the absolute distance. Per-participant means were averaged across trials. Four panels, one per metric: RMSE(Δv_G), RMSE(GTD), RMSE(Δv_U), RMSE(UTD). Bars: means ± SEM across participants; gray for Constant (cohort E_4, $n = 40$; descriptive only, between-cohort) and blue for the three time-varying profiles (cohort E_1, $n = 40$; Linear, Sinusoidal, Semicircular). Panel titles report the omnibus 1-way RM-ANOVA across the three time-varying profiles: $F_{(2,78)} = 4.30$, $p = 0.017$, partial $\eta^2 = 0.099$ for RMSE(Δv_G); $F_{(2,78)} = 40.59$, $p < 0.001$, partial $\eta^2 = 0.510$ for RMSE(GTD); $F_{(2,78)} = 49.81$, $p < 0.001$, partial $\eta^2 = 0.561$ for RMSE(Δv_U); $F_{(2,78)} = 70.37$, $p < 0.001$, partial $\eta^2 = 0.643$ for RMSE(UTD). Pairwise contrasts (Bonferroni-corrected within metric) appear in the main-text **Results**. The Constant cohort is shown for descriptive comparison only and was not included in the within-subject ANOVA.

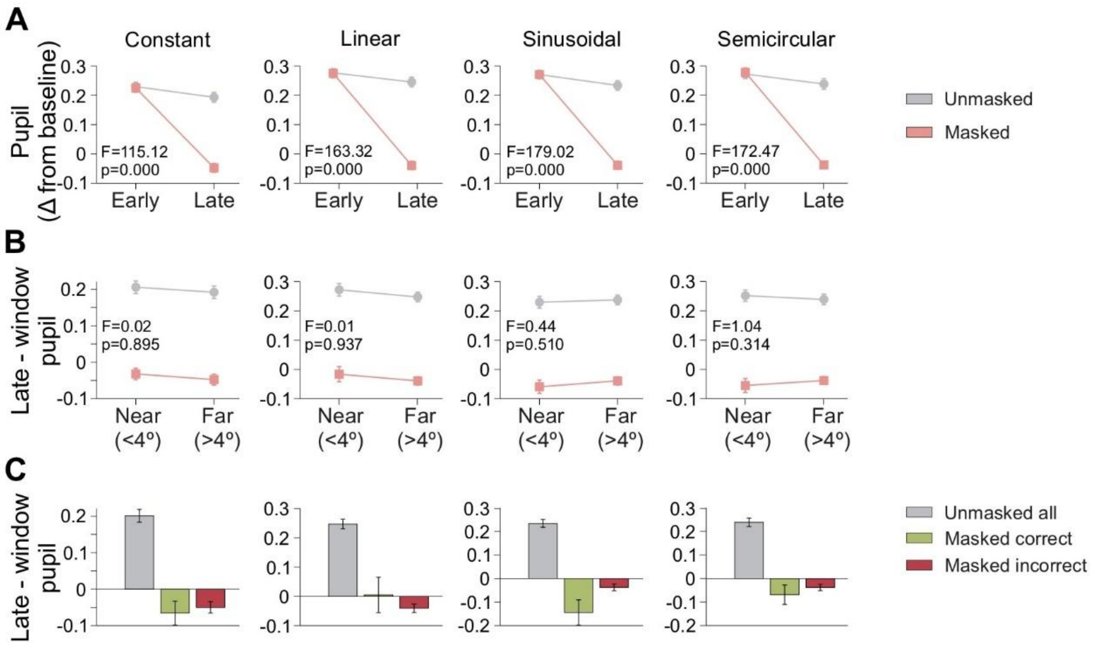

Supplementary Figure S11. Pupillometry re-analysis with a 2 × 2 (Window × Visibility) design and a luminance-confound check. (A) Per-profile interaction plots: pupil size (Δ from per-trial baseline) in the early (frames 131–150) and late (frames 281–300) windows for unmasked (gray) and masked (red) trials. Error bars: SEM ($n = 40$ per profile). Window × Visibility F-values are shown in each panel title. (B) Gaze-locus split: late-window pupil size in unmasked vs masked trials, separated by whether gaze was within 4° of the (still-tracked) target position ("Near") or farther ("Far"). The non-significant Visibility × GazeLocus interaction across all profiles rules out a local-luminance account. (C) Outcome split: late-window pupil size for unmasked-all, masked-correct, and masked-incorrect trials per profile. Incorrect-trial reductions are larger than correct-trial reductions, consistent with internal-estimate gain modulation.

Supplementary Table S1. Participants, cohorts, and trial counts. Four cohorts of 40 participants each (160 total): cohort E_1 received time-varying speed profiles under tonic (full-trial) masking; cohort E_2 received time-varying profiles under phase-locked masking at 0°; cohort E_3 received time-varying profiles under phase-locked masking at 180°; cohort E_4 received the constant-speed condition (with masking). All manipulations (speed profile, modulation frequency, phase, masking) were within-subject and interleaved trial-by-trial. Exemplar trials selected for **Figure 1C** are listed in the second sub-table.

**Cohort**	**Profile**	**n participants**	**Trials/participant**	**Total trials**
Constant v_T	Constant	40	780	31,200
Varying v_T	Linear	40	240	9,600
Varying v_T	Sinusoidal	40	240	9,600
Varying v_T	Semicircular	40	240	9,600
—	All combined	—	—	60,000

Sample trials selected for **Figure 1C** (one per profile and visibility).

**Profile**	**Visibility**	**Trial #**	**CT (s)**	**Source file**
Constant	Unmasked	179	3.38	ID_145_exp_001_v55_ET_p1_A.mat
Constant	Masked	179	3.54	ID_086_exp_001_v55_ET_p2_A.mat
Linear	Unmasked	89	3.87	ID_056_exp_001_v55_ET_p1_A.mat
Linear	Masked	88	3.21	ID_002_exp_001_v55_ET_p1_A.mat
Sinusoidal	Unmasked	167	3.73	ID_049_exp_001_v55_ET_p3_A.mat
Sinusoidal	Masked	236	3.17	ID_066_exp_001_v55_ET_p3_A.mat
Semicircular	Unmasked	214	3.03	ID_002_exp_001_v55_ET_p1_A.mat
Semicircular	Masked	83	3.84	ID_030_exp_001_v55_ET_p3_A.mat

Supplementary Table S2. Collision-time distributions and trial accounting per profile and visibility. Per cell, total trials, successful trials, percentage of successful interceptions, mean and standard deviation of collision time (CT, in seconds) on successful trials, and (masked only) the fraction of successful interceptions that completed before the 2.5 s masking onset. These pre-mask trials are unaffected by visual manipulation and are excluded from the masked-window analyses. Overall success rate across the full dataset: 66.0% (*n* = 60,000 trials). Companion to **Supplementary Figure S1**.

Profile	Vis.	n trials	Success %	CT mean (s)	CT SD (s)	CT median (s)	CT < 2.5 s
Constant	Unm	15,600	75.8	2.04	1.18	1.78	—
Constant	Msk	15,600	63.7	1.69	0.94	1.50	82.5%
Linear	Unm	4,800	69.3	2.28	1.29	2.09	—
Linear	Msk	4,800	54.0	1.77	0.99	1.62	79.3%
Sinusoidal	Unm	4,800	68.8	2.22	1.26	1.98	—
Sinusoidal	Msk	4,800	55.0	1.77	1.00	1.62	79.5%
Semicircular	Unm	4,800	71.3	2.20	1.25	1.98	—
Semicircular	Msk	4,800	53.4	1.76	1.01	1.59	79.0%

Supplementary Table S3. Saccade-exclusion sensitivity of the masked-versus-unmasked contrast on gaze velocity and gaze–target distance. Side-by-side comparison of paired t-test results on Δv_G and GTD with and without saccade-like frames excluded. Saccade frames: $|v_G| > 100°/s$ with ±1-frame padding; trials with more than 50% saccade frames in the 2.5–5 s analysis window were excluded from the saccade-excluded version. Pearson r values reflect the participant-level correlation between original and saccade-excluded per-participant means, pooled across visibility. $n = 40$ participants per profile. All masked-versus-unmasked contrasts are significant at $p < 0.001$ in both versions; effect sizes (*dz*) are larger after saccade exclusion. Companion to **Supplementary Figure S2**.

Δv_G (gaze speed error, °/s)

**Profile**	**Version**	**Unm mean**	**Msk mean**	$\|t_{(39)}\|$	***dz***	**Pearson *r***
Constant	Original	−7.45	−13.30	7.57	−1.20	0.46
Constant	Sacc-excl.	−22.00	−27.45	10.61	−1.68	—
Linear	Original	−2.39	−6.18	4.65	−0.74	0.52
Linear	Sacc-excl.	−15.08	−20.14	13.26	−2.10	—
Sinusoidal	Original	−2.68	−7.21	4.90	−0.77	0.53
Sinusoidal	Sacc-excl.	−15.66	−20.66	11.61	−1.84	—
Semicircular	Original	−2.64	−7.78	5.95	−0.94	0.54
Semicircular	Sacc-excl.	−15.85	−21.40	13.49	−2.13	—

GTD (gaze-target distance, °)

**Profile**	**Version**	**Unm mean**	**Msk mean**	$\|t_{(39)}\|$	***dz***	**Pearson *r***
Constant	Original	9.56	10.52	5.11	0.81	0.993
Constant	Sacc-excl.	9.48	10.54	5.46	0.86	—
Linear	Original	7.81	10.90	27.75	4.39	0.995
Linear	Sacc-excl.	7.49	10.91	31.13	4.92	—
Sinusoidal	Original	8.05	10.78	19.49	3.08	0.993
Sinusoidal	Sacc-excl.	7.77	10.78	21.04	3.33	—
Semicircular	Original	8.42	10.84	18.98	3.00	0.993
Semicircular	Sacc-excl.	8.20	10.85	20.15	3.19	—

Supplementary Table S4. Per-speed masking effects under constant target velocity. Number of velocity bins (out of 13: 5, 10, 15, …, 65°/s) at which masked versus unmasked trials differed after FDR correction (Benjamini–Hochberg across the 13 tests within each metric). The right column lists the specific velocities at which the contrast did not survive FDR correction. Manual speed error (Δv_U) shows the most restricted masking effect, significant only at the higher target velocities. Companion to **Supplementary Figure S4**.

**Metric**	**FDR-significant speeds**	**Non-significant velocities (°/s)**
Δv_G	12 / 13	25
GTD	11 / 13	45, 50
Δv_U	4 / 13	5, 10, 15, 20, 25, 30, 35, 40, 50
UTD	13 / 13	—

Supplementary Table S5. Acceleration versus deceleration at matched instantaneous target velocity. Per-bin paired contrasts comparing behavior at matched instantaneous v_T between the acceleration and deceleration phases of the linear-ramp condition. Per metric: number of velocity bins surviving FDR correction (Benjamini–Hochberg across the 10 bins), number of bins with Bayes factor BF01 > 3 (positive evidence for the null; JZS Cauchy prior, $r = \sqrt{2}/2$), and summary of the effect direction. v_T bin centers: 12.5, 17.5, 22.5, 27.5, 32.5, 37.5, 42.5, 47.5, 52.5, 57.5°/s. Unmasked trials only, cohort E_1, $n = 40$ participants. Companion to **Supplementary Figure S5**.

**Metric**	**FDR-significant bins**	**BF_{01} > 3 (null support)**	**Effect direction**
Δv_G	10 / 10	0 / 10	Phase effect at all bins
GTD	10 / 10	0 / 10	Phase effect at all bins
Δv_U	10 / 10	0 / 10	Phase effect at all bins
UTD	10 / 10	0 / 10	Phase effect at all bins
